# Species delimitation in frogs from South American temperate forests: The case of *Eupsophus*, a taxonomically complex genus with high phenotypic variation

**DOI:** 10.1371/journal.pone.0181026

**Published:** 2017-08-15

**Authors:** Claudio Correa, Dayana Vásquez, Camila Castro-Carrasco, Álvaro Zúñiga-Reinoso, Juan Carlos Ortiz, R. Eduardo Palma

**Affiliations:** 1 Departamento de Zoología, Facultad de Ciencias Naturales y Oceanográficas, Universidad de Concepción, Barrio Universitario S/N, Concepción, Chile; 2 Departamento de Ecología, Facultad de Ciencias Biológicas, Pontificia Universidad Católica de Chile, Alameda 340, Santiago, Chile; 3 Facultad de Medicina Veterinaria, Universidad San Sebastián, Lientur 1457, Concepción, Chile; 4 Departamento de Ciencias Ecológicas, Facultad de Ciencias, Universidad de Chile, Las Palmeras 3425, Santiago, Chile; Laboratoire de Biologie du Développement de Villefranche-sur-Mer, FRANCE

## Abstract

One of the most characteristic and abundant amphibian taxa of South American temperate forests is *Eupsophus*. The ten currently recognized species of the genus have been divided in two species groups, *roseus* and *vertebralis*, but most of them, eight, belong to the *roseus* group. Recent phylogeographic and phylogenetic studies have suggested that species diversity of the *roseus* group could be underestimated. An examination of the literature shows that species of the *roseus* group exhibit high levels of variation in their external characteristics, particularly those used as diagnostic characters, which compromises their taxonomy and hinders their field recognition. High levels of variation were also observed in several new populations of the *roseus* group discovered in southern Chile (36°-40°S), which could not be identified to the species level by their external characteristics. On the other hand, the literature reveals a scarse karyotype differentiation and a high bioacoustic uniformity among the species of the *roseus* group. We performed a Bayesian phylogenetic analysis using mitochondrial and nuclear genes to reevaluate the species diversity of the *roseus* group, including all the nominal species of *Eupsophus* and new populations. This analysis was complemented with three species delimitation approaches, General Mixed Yule Coalescent, multi-rate Poisson Tree Process and Automatic Barcode Gap Discovery. We favored a conservative delimitation of only four species for the *roseus* group, a result more consistent with the distribution of pairwise genetic distances, and the available chromosome and bioacoustic evidence. The four recognized lineages, which have nearly completely allopatric distributions, are named after the earliest nominal species that they include, but because high levels of phenotypic variation, they are not diagnosable by consistent differences in external morphology. We discuss the implications of this new proposal for the taxonomy and conservation of the genus, and the possible causes of the difficulty to estimate its species diversity.

## Introduction

The steady increase in the number of amphibian species is largely explained by an intensified exploration of tropical areas, particularly of megadiverse countries [1], where the application of techniques such as bioacoustics and molecular genetics has revealed high levels of cryptic diversity (e.g. [2–7]). In contrast, temperate regions have relatively fewer amphibian species [8], so it is expected that explorations and the associated taxonomic work in these regions contribute much less to the growing global amphibian inventory. Effectively, an examination of the geography of amphibian descriptions in the last five years shows that, with very few exceptions, most of the new species have been discovered in tropical zones [9]. However, despite of the paucity of descriptions in temperate regions, some of the discoveries suggest that part of their amphibian diversity is due to the presence of cryptic species (e.g. [10–12]).

Notwithstanding their low species richness, temperate regions harbor a great diversity in terms of evolutionary history [13], as exemplified by the amphibians from temperate forests and surrounding Patagonian environments of southern South America (Chile and Argentina). This evolutionarily heterogeneous group of anurans [14, 15] is currently comprised by about 52 species belonging to thirteen genera and six families [16, 17], but only three genera, *Alsodes*, *Atelognathus* and *Eupsophus*, account for more than half of the species. Only *Eupsophus*, with ten currently recognized species [18, 19], is found exclusively in the temperate forests, inhabiting mainly the forest ground [14].

**Fig 1:**
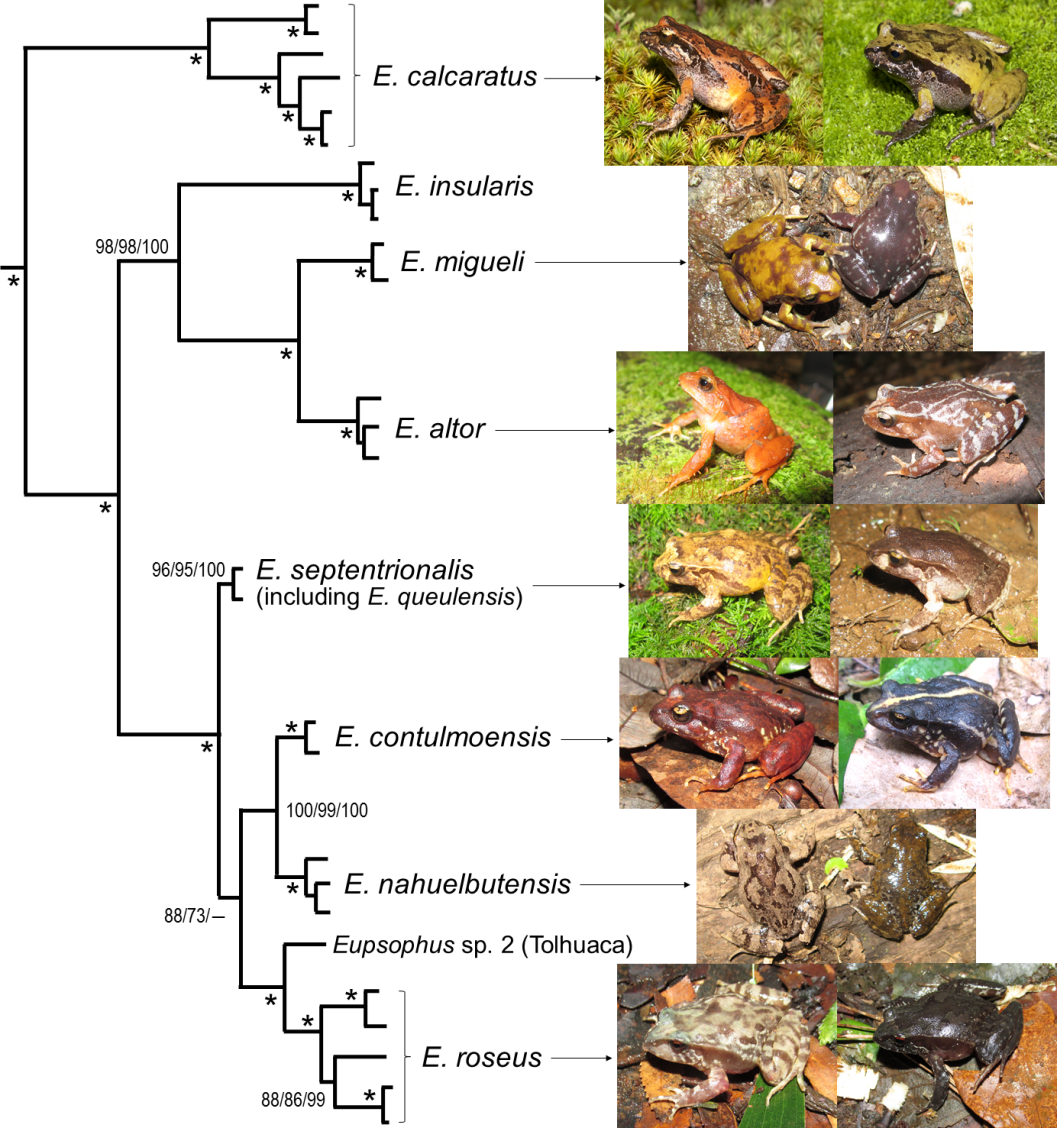
Phylogenetic relationships after Blotto et al. ([18], their Fig 2) and variation of body coloration of the *Eupsophus roseus* group. The tree depicts the relationships among the eight currently recognized species of the group plus a probable undescribed species (sp. 2) from Tolhuaca, Chile, according to the parsimony analysis of Blotto et al. [18]. Numbers along nodes separated by slashes indicate jackknife values of the two different maximum parsimony analyses and posterior probabilities of the Bayesian analysis of Blotto et al. [18]; asterisks indicate maximum values of jackknife and posterior probabilities; the hyphen denotes a group not recovered in their analyses. The relative lengths of the branches of the original figure were maintained. Pictures exemplify intrapopulation variation of body coloration in specimens from the type localities (excepting *E*. *insularis*): Chiloé Island (*E*. *calcaratus*), Mehuín (*E*. *migueli*), Parque Oncol (*E*. *altor*), R.N. Los Queules (*E*. *septentrionalis*), M.N. Contulmo (*E*. *contulmoensis*), P.N. Nahuelbuta (*E*. *nahuelbutensis*) and Valdivia (*E*. *roseus*) (see maps of Fig A in S5 File).

**Fig 2:**
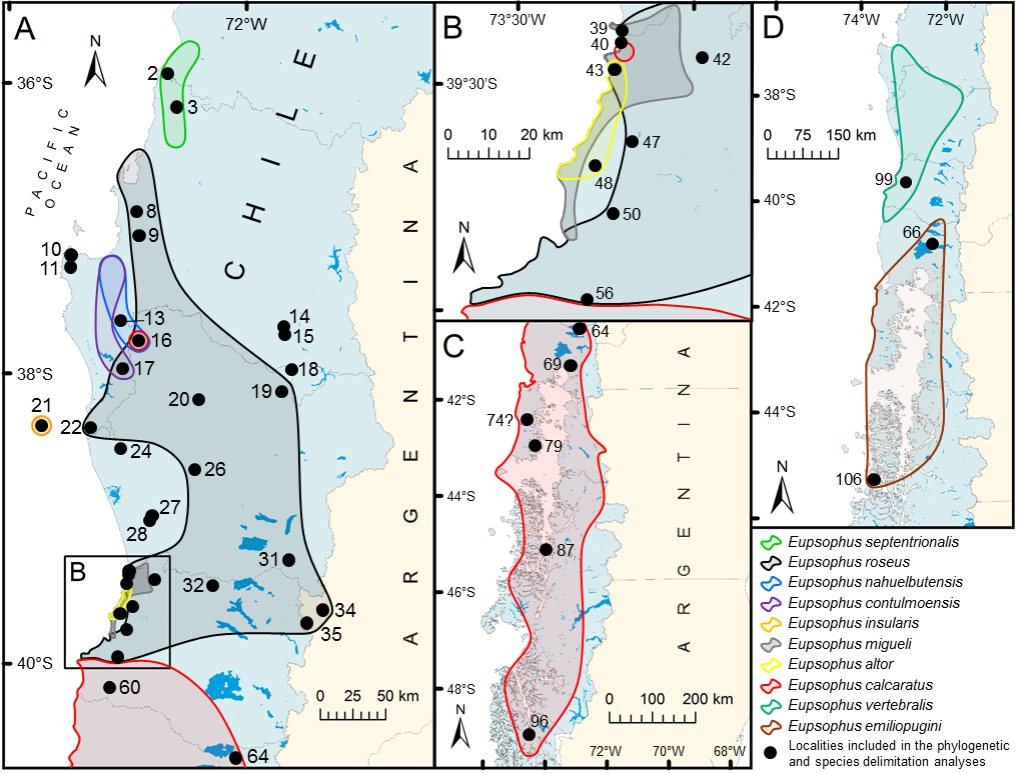
Schematic representation of distribution limits of *Eupsophus* spp. according to the literature. Shaded colored areas represent the distribution limits of the ten species of the genus (maps A, B, and C, *roseus* group; D, *vertebralis* group), which were obtained joining the records compiled from the literature (S5 File). These areas are not intended to depict the real distribution ranges, but only to show the described limits and degree of overlapping among species, mainly in Chile, where the greatest diversity of species is found (see details in S4 File). Inversely, zones not covered by the shaded areas do not mean the absence of the genus, but just the lack of published records. Note the high degree of overlapping among species around 37°50’S (Nahuelbuta Range, localities 16 and 17 of map A) and in the coastal area between 39°22’ and 39°52’S (map B), where even two isolated localities of *E*. *calcaratus* are found (red shaded circles). Only the localities included in the phylogenetic/delimitation analyses are indicated (black circles), and for Argentina, only the two localities of *E*. *roseus* of Blotto et al. [18] included in these analyses were considered. Thin gray lines within Chile represent boundaries of Administrative Regions.

Our understanding of the phylogenetic relationships, evolution and biogeography of *Eupsophus* has significantly improved with the study of Blotto et al. [18] (Fig 1). They ratified the deep division of *Eupsophus* into two groups, *roseus* (*E*. *roseus*, *E*. *calcaratus*, *E*. *insularis*, *E*. *migueli*, *E*. *contulmoensis*, *E*. *nahuelbutensis*, *E*. *septentrionalis* and *E*. *altor*) and *vertebralis* (*E*. *vertebralis* and *E*. *emiliopugini*), which is based on morphological [20, 21], bioacoustic (advertisement calls) [22, 23], chromosome [24], genetic [25], immunological [26], and molecular phylogenetic evidence [15, 18, 21]. The genus ranges mainly in southern Chile (35°50’-49°25’S) and marginally in Argentina, but most species (seven) can be found in the Coastal Range of Chile between 36° and 40°S (an eighth is endemic of Isla Mocha, an island located at 38°22’S, 30 km off the coast) (Fig 2). Blotto et al. [18] suggested that the origin of *Eupsophus* was at the west side of the Andes Mountains and noted that the diversity and distribution of their species are consistent with hypothesized glacial forest refugia in Chile (reviewed in [27]).

The taxonomy of *Eupsophus* has been considered complex [18, 21]. Although Blotto et al. [18] included several specimens of all the nominal species of *Eupsophus* in their phylogenetic analyses and obtained strong support for the inferred relationships, some taxonomic problems persist within the *roseus* group. One of these problems is the specific identity of some populations from Argentina, which alternatively have been assigned to *E*. *roseus*, *E*. *calcaratus*, and again to *E*. *roseus* ([28], discussed in [18]). On the other hand, species level lineages have been identified in the foothills of the Andes of Chile using molecular approaches: one from the locality of Villarrica (39°20’S), considered initially as *E*. *calcaratus* [29], and another from P.N. Tolhuaca (38°13’S), which would correspond probably to an undescribed taxon closely related to *E*. *roseus* [18] (Fig 1). But it can be argued that the most important unresolved problem in the *roseus* group is the taxonomic and geographic delimitation of *E*. *roseus*. Four narrow-range species (*E*. *migueli*, *E*. *contulmoensis*, *E*. *nahuelbutensis* and *E*. *altor*) have been described in Chile within the wider distribution range accepted for *E*. *roseus* in the literature (between 36°50’ and 39°50’S [14, 30]), although still it is unclear if the new taxa are sympatric with this species. In fact, there are reports of syntopy among *E*. *roseus* and three of those four species in their type localities (e.g. [31–33]), but these records have been subsequently ignored (see details in S4 File). Furthermore, the geographic limits between this species and *E*. *calcaratus* in Chile and Argentina (see above) are difficult to define because some specimens of *E*. *roseus* can be mistaken for *E*. *calcaratus* and vice versa, when considering some external features [31].

Recently, it has been suggested that species richness of amphibians in the temperate forests of Chile has been underestimated probably due to the presence of overlooked cryptic species with smaller geographic ranges [34, 35]. The description of species within the geographic range of *E*. *roseus* and the proposal of two candidate species in the genus based on molecular evidence [18, 29] mentioned above are consistent with this scenario. Moreover, the high levels of variation described in body coloration, snout profile, iris color and even osteological features (xiphisternum shape) in several species of the *roseus* group (e.g. [21, 36]; see examples in S1 Fig) might reflect the undetected coexistence of more than one species in some areas. An alternative (albeit not mutually exclusive) explanation is that the high levels of phenotypic variation might represent true intraspecific polymorphisms, a possibility rarely raised explicitly in the literature [34]. Regardless, the genus *Eupsophus* could be a good model to evaluate if South American temperate forests harbor high levels of species richness and cryptic diversity of anurans as tropical environments.

In this study, we reassess the species diversity of the *roseus* group to clarify its taxonomy, the degree of sympatry among its species, and the status of the two proposed candidate species. The reassessment was based on the most comprehensive molecular phylogenetic analysis of the genus to date, which included all the recognized species and undescribed populations of the *roseus* group, as well as three species delimitation approaches. The undescribed populations mostly came from the distribution range of *E*. *roseus* in southern Chile (36°-40°S), but they could not be identified at the species level by external characteristics. We also compiled the main types of evidence used in the taxonomic and systematic studies of *Eupsophus*, with an emphasis in the characters included in the diagnoses, to reevaluate the evidential basis of its current taxonomy. This review was complemented with a compilation of geographic distribution data, to detect problems in the geographic delimitation of the species. Based on the phylogenetic and species delimitation analyses, we propose an estimate of species level lineages within the *roseus* group, which also allows us to address the most important taxonomic and biogeographic problems of the *roseus* group that arise from the literature.

## Materials and methods

### Ethics statement

Protocols for handling, collection and euthanasia of specimens and this study were approved by the Bioethics and Biosecurity Committees of the Pontificia Universidad Católica de Chile (resolution of August 2012) and Universidad de Concepción (April 2013). All voucher specimens were euthanized by immersion in buffered benzocaine (100 mg/L). The permit for the capture and collection of the animals was provided by the Servicio Agrícola Ganadero (SAG) (resolution 6840/2012).

### Literature sources

For the taxonomy and systematics overview (see Supporting Information), we fundamentally considered the original descriptions and redescriptions, all studies with a primarily taxonomic focus (particularly those using of chromosomes and bioacoustic evidence), and the most recent phylogenetic studies and reviews [18, 21, 36]. With “recent” we mean studies done after the current delimitation of the genus (i.e. including only the species from the temperate forests of Chile and Argentina) was embraced in the late seventies (e.g. [37]). Blotto et al. [18] is the most comprehensive molecular phylogenetic study of the genus to date, so we depict their phylogenetic hypothesis in Fig 1. For the geographic distribution overview (see Supporting Information), we contrast the most recent published maps [16, 21, 38] with the cumulative information from diverse studies about the genus or Chilean amphibians to define the distribution ranges (e.g. [18, 29–31, 39–45]).

### Molecular data and phylogenetic analyses

#### Sampling sites and samples included

For the phylogenetic analysis, 60 new samples of the *Eupsophus roseus* group were obtained from the type and new localities (Table 1, Fig 2 and S2 Table); the rest of samples, 27, were obtained from Blotto et al. [18], so representatives of the ten recognized species were included. We included only two of the three samples from Argentina of Blotto et al. [18], both identified in that study as *E*. *roseus*; the specimen of *E*. *calcaratus* (Lago Puelo) was excluded because not all the gene fragments used here are available for that sample. We added samples from some type localities (except for *E*. *insularis*) to those of Blotto et al. [18] for detecting the possible presence of more than one species on those places, as can be inferred from the literature (see S4 File). Most of the new localities fill distribution gaps among historical records and almost all of them are located within the distribution range of *E*. *roseus* according to some sources (e.g. [30]). However, we could not identify the specimens of these localities to species level considering their external morphological characteristics because they display high levels of intra and interpopulation variation in three qualitative characters commonly used in the species descriptions and diagnoses of *Eupsophus*: dorsal and ventral color patterns, iris color, and lateral and dorsal snout profile (see some examples in S2 Fig). Specimens from Camino a P.N. Villarrica were considered as representatives of the locality of Villarrica, where a “species lineage” related to *E*. *calcaratus* was detected by Nuñez et al. [29] (they did not specify the exact coordinates of that place).

**Table 1:** Localities included in the phylogenetic analysis.

Locality	Latitude (S)	Longitude (W)	Source	Nominal species	Proposed species
**R.N. Los Queules (2)**	**35°59’16”**	**72°41’34”**	**[18], this study**	***E*. *septentrionalis*, *E*. *queulensis***^**a**^	***E*. *roseus***
Cerro El Guanaco* (3)	36°13’22”	72°37’36”	This study	*Eupsophus* sp.	*E*. *roseus*
Cerros de Chiguayante* (8)	36°56’08”	73°00’04”	This study	*Eupsophus* sp.	*E*. *roseus*
Santa Juana* (9)	37°06’08”	72°59’30”	This study	*Eupsophus* sp.	*E*. *roseus*
Llico* (10)	37°12’58”	73°34’57”	This study	*Eupsophus* sp.	*E*. *roseus*
Quidico* (11)	37°17’53”	73°35’31”	This study	*Eupsophus* sp.	*E*. *roseus*
Rucapehuén (13)	n.i.	n.i.	[18]	*E*. *nahuelbutensis*	*E*. *roseus*
Alto Biobío* (14)	37°45’54”	71°46’23”	This study	*Eupsophus* sp.	*E*. *roseus*
Loncopangue* (15)	37°49’08”	71°45’59”	This study	*Eupsophus* sp.	*E*. *roseus*
**P.N. Nahuelbuta (16)**	**37°48’05”**	**73°00’15”**	**[18], this study**	***E*. *nahuelbutensis***	***E*. *roseus***
**M.N. Contulmo (17)**	**38°00’47”**	**73°11’05”**	**[18], this study**	***E*. *contulmoensis***	***E*. *roseus***
Pemehue* (18)	38°03’47”	71°43’07”	This study	*Eupsophus* sp.	*E*. *roseus*
Tolhuaca^b^ (19)	n.i.	n.i.	[18]	*Eupsophus* sp. 2	*E*. *roseus*
Río Traiguén* (20)	38°14’54”	72°31’19”	This study	*Eupsophus* sp.	*E*. *roseus*
**Isla Mocha (21)**	**n.i.**	**n.i.**	**[18]**	***E*. *insularis***	***E*. *insularis***
Primer Agua^c^ (22)	38°24’38”	73°29’25”	This study	*Eupsophus* cf. *roseus*	*E*. *insularis*
Camino a Villa Las Araucarias* (24)	38°33’40”	73°13’47”	This study	*Eupsophus* sp.	*E*. *insularis*
M.N. Cerro Ñielol (26)	38°43’35”	72°35’24”	This study	*E*. *roseus*	*E*. *roseus*
Santa Amelia* (27)	39°02’04”	72°59’03”	This study	*Eupsophus* sp.	*E*. *roseus*
Pumalal* (28)	39°03’47”	73°00’35”	This study	*Eupsophus* sp.	*E*. *roseus*
Camino a P.N. Villarrica*^d^ (31)	39°22’28”	71°47’23”	This study	*Eupsophus* sp.	*E*. *roseus*
Queule (39)	n.i.	n.i.	[18]	*E*. *migueli*	*E*. *migueli*
**Mehuín (40)**	**39°25’44”**	**73°12’41”**	**[18], this study**	***E*. *migueli***	***E*. *migueli***
Puringue* (42)	39°28’19”	72°58’50”	This study	*Eupsophus* sp.	*E*. *roseus*
Alepúe (43)	n.i.	n.i.	[18]	*E*. *roseus*	*E*. *roseus*
Malalhue* (32)	39°31’53”	72°28’19”	This study	*Eupsophus* sp.	*E*. *roseus*
San Martín (47)	n.i.	n.i.	[18]	*E*. *roseus*	*E*. *roseus*
**Parque Oncol (48)**	**39°41’54”**	**73°18’06”**	**[18], this study**	***E*. *altor***	***E*. *migueli***
Lago Paimún, Argentina (34)	n.i.	n.i.	[18]	*E*. *roseus*	*E*. *roseus*
Termas de Epulafquén, Argentina (35)	n.i.	n.i.	[18]	*E*. *roseus*	*E*. *roseus*
**Valdivia (50)**	**39°48’16”**	**73°15’27”**	**This study**	***E*. *roseus***	***E*. *roseus***
Llancahue (99)	n.i.	n.i.	[18]	*E*. *vertebralis*	*E*. *vertebralis*
Naguilán* (56)	39°59’37”	73°20’40”	This study	*Eupsophus* sp.	*E*. *roseus* and *E*. *calcaratus*
Camino a P.N. Alerce Costero* (60)	40°11’48”	73°25’56”	This study	*E*. *calcaratus*	*E*. *calcaratus*
Puyehue (64)	n.i.	n.i.	[18]	*E*. *calcaratus*	*E*. *calcaratus*
**La Picada (66)**	**n.i.**	**n.i.**	**[18]**	***E*. *emiliopugini***	***E*. *emiliopugini***
**Río Correntoso (69)**	41°26’48”	72°39’54”	This study	***E*. *calcaratus***	***E*. *calcaratus***
**Chiloé (74?)**^**e**^	**n.i.**	**n.i.**	**[18]**	***E*. *calcaratus***	***E*. *calcaratus***
Yaldad (79)	n.i.	n.i.	[18]	*E*. *calcaratus*	*E*. *calcaratus*
Isla Chaculay (87)	n.i.	n.i.	[18]	*E*. *calcaratus*	*E*. *calcaratus*
Isla Rivero (106)	n.i.	n.i.	[18]	*E*. *emiliopugini*	*E*. *emiliopugini*
Puerto Edén (96)	n.i.	n.i.	[18]	*E*. *calcaratus*	*E*. *calcaratus*

Nominal species refers to the original labels of the samples or the identification according to the literature. Source indicates if the sequences were obtained in this study and/or were gathered from [18]. Proposed species refers to the nomenclatural changes derived from this study. Numbers in parentheses refer to the maps of Fig 2 and Fig A in S5 File; type localities are in bold; n.i. = not indicated in [18].

*Indicates new localities.

^a^Our material is labeled as *E*. *queulensis*, while that of Blotto et al. [18] as *E*. *septentrionalis* (see S2 Table).

^b^Blotto et al. [18] suggest that a probable new species is found there, previously identified as *E*. *roseus* (e.g. [21]).

^c^Webb & Greer [39] recorded the presence of *E*. *roseus* 7 km SSE from Tirúa, which corresponds almost exactly to the location of Primer Agua.

^d^We considered this locality as representative from the area where Nuñez et al. [29] detected a new species lineage related to *E*. *calcaratus*.

^e^Chiloé refers to a large island which is the imprecise type locality of *E*. *calcaratus*.

#### DNA extraction and sequencing

Two types of tissue were used for obtaining DNA: buccal mucosa and tongue muscle. Buccal mucosa was obtained with swabs Copan 516CS01, which posteriorly were dried with silica gel. Individuals sampled with this method were released immediately in the field after being measured and photographed. Some individuals by locality were selected as vouchers and deposited in the Colección de Flora y Fauna Prof. Patricio Sánchez Reyes (SSUC) of the Departamento de Ecología, Pontificia Universidad Católica de Chile, and in the Museo de Zoología of the Universidad de Concepción (MZUC) (see details in S2 Table). A small piece of tongue was removed from these specimens for DNA extraction. DNA from both types of tissue was extracted with the kit Promega Wizard SV Genomic DNA Purification System. For the phylogenetic analysis, mitochondrial (a fragment which extends between ribosomal genes 12S and 16S, including the tRNA-Val (12S-16S)), and nuclear (fragments of rhodopsin exon 1 (rhod) and Seven in Absentia homolog I (SINA)) genes were used. Primer sequences and PCR conditions for amplifying the 12S-16S fragment (primers 16Sbr-H, 16Sar-L, 1216H, 1216LN, H1478, L1091) are detailed in [15, 46, 47]. Primer sequences for amplifying nuclear fragments were obtained from [48] (rhod), and [49] (SINA). PCR conditions for amplifying nuclear genes were described by Charrier et al. [50]. All mitochondrial and nuclear PCR products were sequenced in both directions in an ABI3730XL automatic sequencer. Sequences were edited with the program BioEdit v7.1.3 [51] and then aligned with Muscle v3.8.31 [52]. Sequences were deposited in GenBank (fragment 12S-16S: accessions numbers KY826236-KY826295; rhod: KY826296-KY826355; SINA: KY826356-KY826415; see S2 Table).

#### Phylogenetic analysis

Phylogenetic relationships among *Eupsophus* specimens were estimated using a Bayesian inference (BI) with Markov Chain Monte Carlo (MCMC) method, performed with the program MrBayes v3.2.1 [53]. We initially selected the nucleotide sequence evolution models for each fragment (mitochondrial fragment 12S-16S, and the two nuclear ones, rhod and SINA) with jModeltest v2.1.10 [54], but decided to use a more flexible approach, reversible-jump, which allows exploring the space of all General Time Reversible sub-models [55]. Thus, reversible-jump, plus gamma and proportion of invariable sites parameters (also selected by jModeltest), was applied independently to each partition (fragment). Two independent analyses (each consisting of two groups of four chains that run independently) applying that method were run for 20 million generations, sampling every 1,000th generation. The first 25% of generations were conservatively discarded as burn-in after observing the stationarity of ln-likelihoods of trees in Tracer v1.6 [56]. Convergence and mixing of chains was assessed examining values of average standard deviation of split frequencies (ASDSF), and expected sampling sizes (ESS) and Potential Scale Reduction Factor (PSRF) for all parameters. Trees were rooted with one specimen of *Alsodes barrioi* (GenBank accession numbers JX204153, JX204089 and JX204224; obtained from [18]).

### Species delimitation analyses

We assess the taxonomic status of the populations of the *roseus* group included in the phylogenetic analysis applying three species delimitation methods, General Mixed Yule Coalescent (GMYC, [57]), multi-rate Poisson Tree Process (mPTP, [58]), and Automatic Barcode Gap Discovery (ABGD, [59]), which are based on different aspects of molecular evolution. GMYC finds the maximum likelihood (ML) solution for a model that combines diversification between species (based on a Yule model) and genealogical branching within species (based on a neutral coalescent model) on a time-calibrated ultrametric tree [60]. On the other hand, PTP methods model intra and interspecies processes by directly using the number of substitutions [61]. The original PTP can determine the transition point among the processes occurring between species and within a species using a two-parameter model, a parameter for speciation and another for coalescent processes, so the adjustment of both parameters delimits the species in a given topology. However, recently a new algorithm based on PTP was implemented, the multi-rate PTP [58], which improves the estimate in phylogenies that have different rates of speciation-coalescence. Although the speciation rate can be assumed as constant between sister species, intraspecific coalescence rate and consequently genetic diversity may vary significantly even among sister species, therefore an analysis like mPTP allows to account for the different rates of branching events within each delimited species [58]. Finally, the Automatic Barcode Gap Discovery (ABGD) method [59] was employed to statistically partition the samples into candidate species based on a barcode gap (i.e. a gap in the pairwise genetic distance distribution, presumably between intraspecific and interspecific distances). We applied this method because in an exploratory analysis of the concatenated mitochondrial and nuclear data set, we observed very clear discontinuities in the distribution of genetic distances that can be interpreted as barcode gaps (see S3 Fig). GMYC and mPTP analyses were performed only with the mitochondrial sequences (fragment 12S-16S) because their variation levels are much higher than those of the nuclear data. For the GMYC analysis, a strictly ultrametric Bayesian tree was constructed with BEAST, under the relaxed clock lognormal model and the Yule process speciation model [62]. Then, we ran the multiple-threshold option of GMYC through its web server (http://species.h-its.org/gmyc). For the mPTP, a ML tree was reconstructed with RAxML [63] using the GTR + I + G model of nucleotide substitution, selected with jModelTest v2.1.10 [54]. To perform the mPTP we use the web server (http://mptp.h-its.org/). Finally, we ran ABGD with the mitochondrial and nuclear data combined, using three distance options (Jukes-Cantor, Kimura 2-parameter and Simple). A range between 0.001 to 0.25 of prior intraspecific divergence values was assayed (in ten steps), applying a relative gap width (X) of 1.5 [59]. This analysis was performed through the web server of ABGD (http://wwwabi.snv.jussieu.fr/public/abgd/abgdweb.html). For all these analyses, sequences of the *vertebralis* group were excluded, but one specimen of *E*. *emiliopugini* from La Picada (Table 1) was used to root ML trees for GMYC and mPTP analyses. Identical sequences were excluded for GMYC and mPTP analyses.

## Results

### Taxonomy and systematics overview

In Supporting Information, we provide a brief summary of the recent taxonomy of the genus, with an emphasis on the characters used to diagnose the species (S1 File). Table A in S1 File summarizes the diagnoses of the species of the *roseus* group, indicating which characters were variable in the type series. The S1 File also provides antecedents showing that the four most frequently included characters in the diagnoses (body coloration, color of the upper part of iris, shape of snout and shape of the distal end of the xiphisternum) vary at the intrapopulation level, even in the type localities of some species (see also S1 Fig). We also synthesized the bioacoustics (S2 File) and chromosome studies (S3 File) focused directly to the taxonomy and systematics of the genus. Those reviews reveal, despite of the taxonomic importance given to these traits (particularly karyotypes), a scarse differentiation among the advertisement calls and inconsistencies among the karyotypes described for the species of the *roseus* group.

### Geographic distribution overview

The geographic data compiled from the literature are summarized in Fig 2 and Fig A in S5 File. In Fig 2, the distribution limits of the ten species of the genus were represented as color shaded areas drawn joining all the records compiled in the Fig A in S5 File. Those figures reveal several instances of syntopy and range overlapping among species of the *roseus* group inferred from the published records, a pattern that contrasts with the information from other sources of geographic data (see details in S4 File). S4 File also highlights some general patterns and problems derived from published geographic data of the genus.

### Taxonomic proposal

#### Phylogenetic analysis

**Fig 3:**
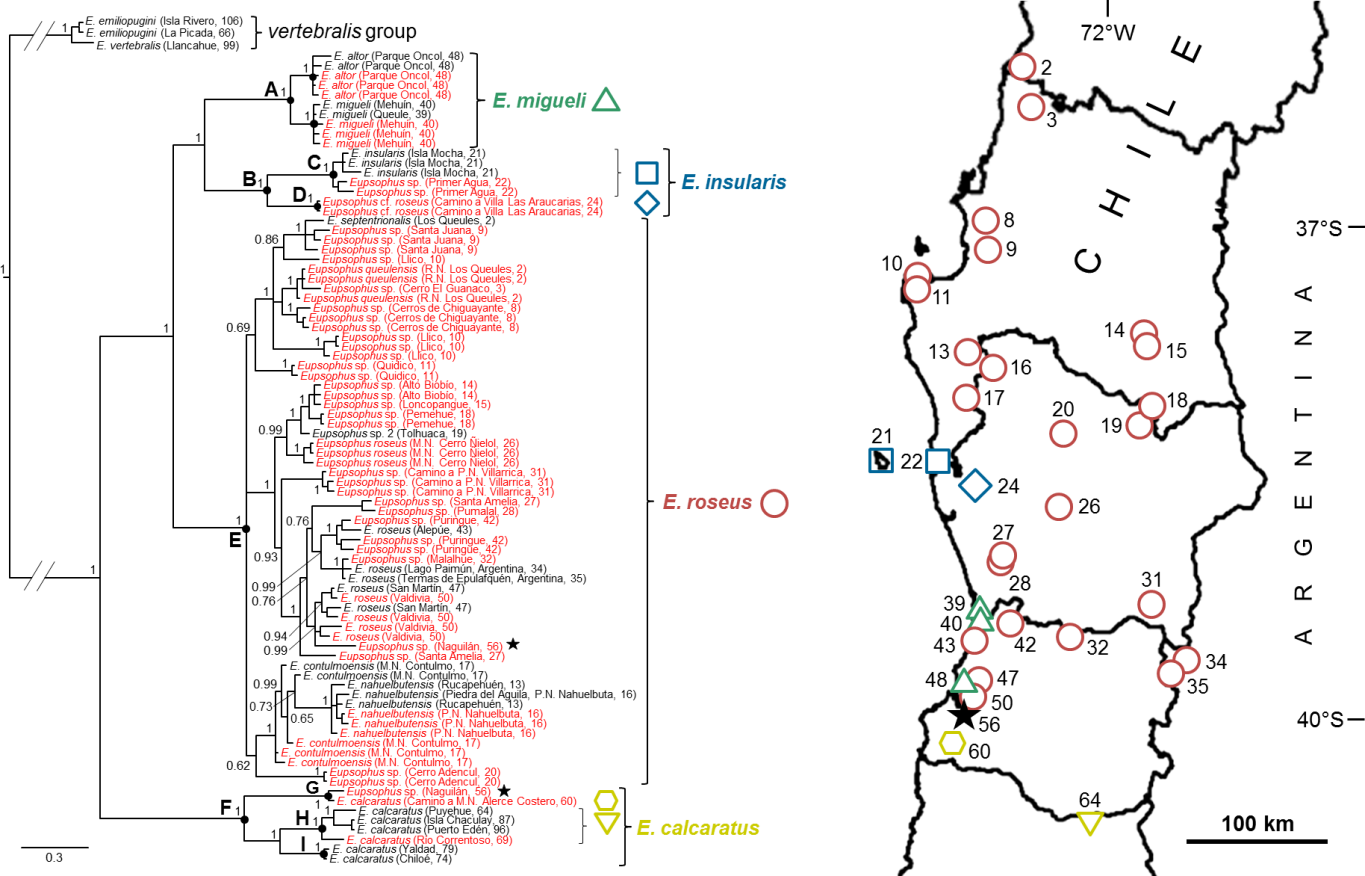
Bayesian consensus tree showing the relationships among the nominal species of *Eupsophus* and new populations of the *roseus* group. The tree was simplified by deleting the outgroup (*Alsodes barrioi*) and shortening the more basal branches (indicated by parallel lines). Samples in red correspond to the specimens added in this study. Numbers along the nodes indicate posterior probability values. The scale bar below the tree represents the expected substitutions per site. Nodes labeled A, C, D, E, G, H, and I indicate the seven candidate species obtained with mPTP and relaxed ABGD analyses (Fig 4); the map on the right shows with different colored symbols the localities corresponding to six of these seven lineages (clade I distributes further south, out of the map). Nodes A, B, E, and F correspond to the result of the conservative ABGD analysis, which is our favored estimate of species of the *roseus* group (localities of the four species depicted with the same colors of the Fig 4). The black stars in the tree indicate the samples of *E*. *roseus* and *E*. *calcaratus* (as identified here) found in syntopy (Naguilán, locality indicated by the star in the map). Dark lines within Chile represent boundaries of Administrative Regions.

We obtained an alignment of 1996 nucleotide sites for the mitochondrial fragment (35 sites with gaps, including *A*. *barrioi*) and 706 additional sites for both nuclear gene fragments. Convergence and mixing of the chains of the Bayesian analysis were achieved according to ASDSF (< 0.0005), ESS (> 1000), and PSRF (0.99998–1.00575) values. The Bayesian consensus tree supported with maximum posterior probability (pp = 1) the two traditionally recognized species groups, *roseus* and *vertebralis* (Fig 3). The relationships among the nominal species of the *roseus* group agree with those obtained by Blotto et al. [18] with their maximum parsimony and Bayesian analyses (Fig 1): *E*. *calcaratus* was recovered as the sister taxon of the rest of species, which in turn grouped in two highly supported clades, one that includes all samples of *E*. *altor*, *E*. *migueli* and *E*. *insularis*, and the other that included *E*. *contulmoensis*, *E*. *nahuelbutensis*, *E*. *roseus*, *E*. *septentrionalis* (one sample from [18]) and our three samples labeled as *E*. *queulensis* (Fig 3). Within the first clade, *E*. *migueli* + *E*. *altor* (node A in the Fig 3) constitutes the sister group of *E*. *insularis* + samples from Primer Agua + Camino a Villa Las Araucarias (B). The second clade (E), which includes most of the samples added in this study, is a polytomy of three groups, two of which have low values of pp (< 70). The first group (pp = 0.69) comprised all the samples from coastal localities from R.N. los Queules (labeled as *E*. *septentrionalis* and *E*. *queulensis*) to Quidico (35°59’-37°18’S). The second group (pp = 0.62) included all samples of *E*. *contulmoensis* and *E*. *nahuelbutensis* (both from the Nahuelbuta Range), and those from the interior locality Río Traiguén (37°40’-38°15’S). The third group, the only one with high support (pp = 1), has the greater geographic distribution and comprises the samples from Valdivia (type locality of *E*. *roseus*), nearby interior and coastal localities, western Andean localities, and both from Argentina (Fig 3).

#### Species delimitation

**Fig 4:**
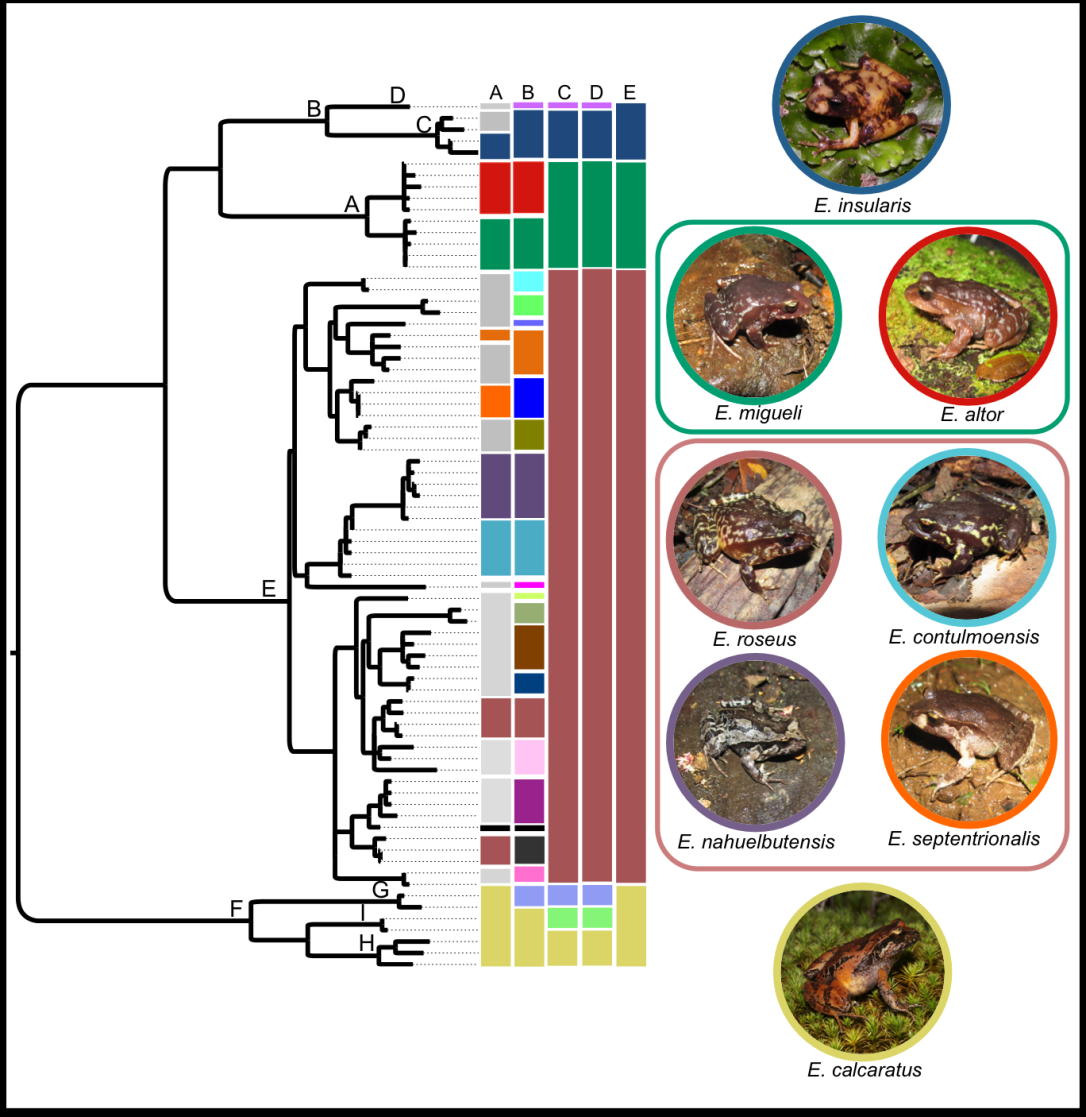
Candidate species of the *Eupsophus roseus* group, according to three species delimitation analyses. Candidate species obtained with each analysis are depicted as colored boxes arranged in columns: General Mixed Yule Coalescent (GMYC, column B), multi-rate Poisson Tree Process (mPTP, column C), Automatic Barcode Gap Discovery (ABGD; columns D, relaxed, and E, conservative results). Groupings obtained with each analysis are contrasted with the current taxonomy (column A) and the maximum likelihood tree used in mPTP. Clades labeled with capital letters are the same of Fig 3. Gray boxes in column A represent the undescribed populations included in this study. Pictures of representatives of the eight previously recognized species of the *roseus* group are bordered with the same colors used in all the columns. Pictures were enclosed according to the favored taxonomic proposal (conservative ABGD). The small black boxes of columns A and B correspond to *Eupsophus* sp. 2 of Blotto et al. [18].

Fig 4 summarizes the candidate species (CSs) of the *roseus* group obtained with GMYC, mPTP and ABGD in comparison with the ML tree (RAxML) and the current taxonomy (see Fig 1). The multiple-threshold GMYC analysis yielded the highest number of subdivisions, suggesting that the *roseus* group would consist of 25 CSs, of which 17 would be new according to the current taxonomy of the group (column A of Fig 4). The number of CSs obtained with mPTP is much lower, seven, only three of which coincide with those obtained by GMYC. In the analysis ABGD, between one and eight CSs were recovered by the initial or recursive partitions, depending on the type of distance and prior intraspecific divergence (P) values, but in general, the number of groups decreased as increased P. We did not consider the solutions with the extreme P values [59], so we present the results of four (which we call conservative) and seven (relaxed) CSs (both depicted in Fig 4). This last result is identical to that obtained with mPTP, so also is very different to that of GMYC (more than triple than the other two methods). This is not unexpected since GMYC shows tendencies for oversplitting in simulated and empirical datasets (e.g. [61, 64–66]), so accordingly we consider as more probable the delimitations of mPTP and ABGD (see below). Regarding the divergence patterns, an analysis of the distribution of pairwise genetic distances of the entire data set (mitochondrial plus nuclear sequences; excluding the samples of *E*. *emiliopugini* and *E*. *vertebralis*) shows two clear barcode gaps, irrespective of the distance assayed, one centered around 0.025 and the other around 0.04 (S3 Fig). The interpretation of the first barcode gap as the intra/interspecies genetic distance limit [59] is only compatible with the arrangement of four CSs (conservative ABGD, Fig 4; see pairwise genetic distances among CSs of relaxed ABGD analysis in S1 Table). Note that this genetic distance limit emerges naturally from the distribution of pairwise genetic distances instead of from a priori threshold value chosen to define between intra and interspecific divergence. On the other hand, the available chromosome and bioacoustic evidence (S2 and S3 Files) support an even lower diversity of species for the *roseus* group: only two divisions defined by the absence/presence of heteromorphic sexual chromosomes, and no division when considering the advertisement calls. Therefore, taking into account the genetic divergence and the chromosome and bioacoustic evidence, we favor the most conservative estimate obtained among all delimitation approaches (conservative ABGD), recognizing only four species within the *roseus* group: *E*. *calcaratus*, *E*. *roseus*, *E*. *migueli* and *E*. *insularis* (Fig 4). All these species correspond to well-supported nodes in the Bayesian consensus tree (Fig 3) and receive their names from the first described nominal species that they include. This proposal entails the synonymy of *E*. *contulmoensis*, *E*. *nahuelbutensis* and *E*. *septentrionalis* with *E*. *roseus*, and that of *E*. *altor* with *E*. *migueli*. Likewise, it implies the range extension of *E*. *insularis* to two continental localities (Primer Agua and Camino a Villa Las Araucarias). Finally, we recognize that two of the methods of delimitation of species (mPTP and relaxed ABGD) suggest seven species for the *roseus* group (Fig 4), but here we opted for the conservative hypothesis for two reasons. First, the chromosomal and bioacoustic evidence available to date suggests no more than two species in the *roseus* group, and second, the two clades containing the additional CSs (*E*. *calcaratus* and *E*. *insularis*, nodes B and F in Figs 3 and 4) were under-sampled in relation to the redefined *E*. *roseus* (node E). We suggest that this geographic sampling bias is influencing the species delimitation process. Thus, the more intense sampling through the distribution of *E*. *roseus* allowed the detection of genetically intermediate lineages, which together shortened the internal branches. Conversely, the relatively sparse sampling within the distribution ranges of *E*. *calcaratus* and *E*. *insularis* could be overestimating the intraspecific divergence among some lineages, so some of them would be recognized as CSs due to their greater branch lengths (e.g. clades D and G, Figs 3 and 4). Therefore, a greater sampling effort is required within *E*. *calcaratus* and *E*. *insularis* ranges to reassess the status of the additional CSs identified within these clades. Following we provide additional information to support the proposed taxonomic changes and point out their biogeographic implications.

#### Delimitation of *E*. *roseus*

The taxonomic and geographic delimitation of *E*. *roseus* must be reevaluated considering the validity of three species described later: *E*. *contulmoensis*, *E*. *nahuelbutensis* and *E*. *septentrionalis*. Our results show that these four nominal species, along with other undescribed populations, have low levels of genetic divergence among themselves, together comprise a well-supported clade in the phylogenetic analysis (node E of Fig 3), and are recognized as a single species by mPTP and ABGD analyses (Fig 4). In the literature, there are additional morphological and genetic antecedents that challenge their taxonomic status. The diagnoses of *E*. *contulmoensis*, *E*. *nahuelbutensis* and *E*. *septentrionalis* [33, 67, 68] include body coloration and that of *E*. *nahuelbutensis* the shape of snout, two characters that show variation in their type series (Table A in S1 File; see also Fig 1) and in other phylogenetically related populations (S1 and S2 Figs). According to the literature, the color of the upper part of the iris is very similar among *E*. *contulmoensis* (bronze-yellow), *E*. *nahuelbutensis* (yellowish) and *E*. *septentrionalis* (light yellow), differing from that of *E*. *roseus* (orange, [41]). However, the coloration of the iris varies in *E*. *roseus*, as mentioned by Nuñez et al. [31] (some specimens have the upper part of the iris bronze-yellow), and as we show here with specimens from the type locality (S1B Fig). Also, we show that this character is very variable within the genus (S1 and S2 Figs). On the other hand, according to their descriptions, *E*. *contulmoensis*, *E*. *nahuelbutensis* and *E*. *septentrionalis* have the end of the xiphisternum rounded. However, Díaz [36] showed in *E*. *roseus* from the type locality (Valdivia) that the shape of the xiphisternum is variable, being rounded the more common condition. There are two allozyme studies involving these species. Ibarra-Vidal et al. [68] found a low level of genetic divergence among *E*. *roseus*, *E*. *contulmoensis*, *E*. *nahuelbutensis* and *E*. *septentrionalis* (although they described two nearly fixed loci to differentiate *E*. *septentrionalis* from *E*. *roseus*). At intraspecific level, Formas et al. [30] found a lower genetic differentiation within *E*. *roseus*, including as part of this species the population from P.N. Nahuelbuta, type locality of *E*. *nahuelbutensis*. Bioacoustic studies (S2 File) agree with the morphological and genetic evidence, showing a lack of differentiaton among *E*. *roseus*, *E*. *contulmoensis*, *E*. *nahuelbutensis* and *E*. *septentrionalis* according to call parameters (Table B in S2 File). The karyotypes also are very similar among *E*. *roseus*, *E*. *contulmoensis* and *E*. *nahuelbutensis* (S3 File), but in *E*. *septentrionalis* heteromorphic sex chromosomes has been described (as *E*. *queulensis*, [69]). This last characteristic is shared with *E*. *migueli* and *E*. *insularis*, two species belonging to other clade according to Blotto et al. [18] and our phylogenetic analysis, but not with *E*. *roseus*, *E*. *contulmoensis* and *E*. *nahuelbutensis* (more related phylogenetically, Fig 3). However, the karyotype data of these species are scarse how to assess the variation of the sex chromosomes morphology in a phylogenetic context. Therefore, taking as a whole our species delimitation analyses and the morphological, bioacoustic, genetic (allozymes and DNA sequences of this study) and phylogenetic evidence (this work), we consider to *E*. *contulmoensis*, *E*. *nahuelbutensis* and *E*. *septentrionalis* as conspecifics of *E*. *roseus*. This redefinition of *E*. *roseus* implies expanding its range to 35°50’S to the north along the coast and to 37°45’S in the foothills of the Andes (new localities 14 and 15, Table 1 and Fig 2). At its southern limit, *E*. *roseus* is replaced by *E*. *calcaratus*, but they are not allopatric as Nuñez et al. [31] proposed, since they are syntopic in Naguilán (39°59’37”S; Fig 3). We also show that populations around Villarrica (originally identified as *E*. *calcaratus*, but considered as a species-level lineage by Nuñez et al. [29]), represented here by Camino a P.N. Villarrica, belongs to *E*. *roseus* (Fig 3).

#### Synonimization of *E*. *altor*

This species was described embracing an integrative taxonomic approach, using morphometric, karyotypic, ethological (advertisement calls and reproductive mode) and molecular data (Nuñez et al. [34]). The new species was compared mainly with *E*. *roseus* and *E*. *migueli*, due to the high morphological similarity and geographical proximity among these species. Nuñez et al. [34] first identified differences in breeding season and tadpole development between the new taxon and *E*. *roseus*, and then added two other diagnostic characters, a call parameter (frequency of spectral elements) and a fixed number of differences in mitochondrial control region sequences. The initial comparison of reproductive traits between *E*. *roseus* and *E*. *altor* is misleading because *E*. *migueli* is the sister species of *E*. *altor* according to the own phylogenetic analysis of Nuñez et al. [34] (relationship ratified by Blotto et al. [18] and this study). The other problem with this reproductive evidence is that the extension of the breeding season and tadpole development still are unknown for *E*. *migueli*. An additional problem arises from the molecular evidence provided by Nuñez et al. [34]. There is a discrepancy between the reported diagnostic divergence between *E*. *altor* and *E*. *migueli* in the paper (nine nucleotide substitutions in the control region) and the true number of differences (22 between both species and seven additional variable sites within *E*. *altor*; GenBank accession numbers JQ780164-JQ780170). Nuñez el at. [29], in a phylogeographic study of *E*. *calcaratus* used sequences of the genes 16S and control region, defining six lineages for this species (labeled A to F). Some specimens of the lineage F differentiate by more than 40 substitutions in the control region sequences (HQ711149-HQ711262), figure that is greater than the actual divergence between *E*. *altor* and *E*. *migueli* (22) of the data set of Nuñez et al. [34]. Therefore, we consider this genetic evidence as insufficient to support the distinction between these two species. Regarding the advertisement calls, Nuñez et al. [34] showed that almost all the parameters measured (call length, fundamental frequency, dominant frequency) have overlapping ranges among *E*. *altor*, *E*. *migueli* and *E*. *roseus* (see also Table B in S2 File). The exception is the maximum frequency of spectral elements, which reaches 20 kHz in *E*. *altor* (in *E*. *migueli* and *E*. *roseus* do not reach 15 kHz, Table 2 of [34]). On the other hand, as shown by Nuñez et al. [34], *E*. *altor* cannot be distinguished morphometrically from *E*. *migueli* and *E*. *roseus*, and has a very similar karyotype to *E*. *migueli* (same fundamental number in females and position of secondary constriction). In summary, there are not enough reproductive data of *E*. *migueli* to compare with *E*. *altor*, the genetic divergence between both species is low as shown by Nuñez et al. [34] and this study, they are morphometrically undistinguishable and have very similar karyotypes, and the advertisement call of *E*. *altor* has the same structure as the other species of the *roseus* group (Table B in S2 File), so the only remaining evidence for diagnosing *E*. *altor* is the maximum frequency of call spectral elements. We consider that this last parameter alone is insufficient to counter all other lines of evidence, therefore we synonymized *E*. *altor* with *E*. *migueli*. According to this taxonomic proposal and literature records, *E*. *migueli* would be distributed between Queule and Los Molinos (39°23’ and 39°51’S approximately, Fig 3).

## Discussion

In this study, a conservative arrangement for the taxonomy of the *Eupsophus roseus* group that reduces from eight to four the species of the group (and to six the species of the genus) is proposed. Although this proposal better reflects all the phylogenetic, genetic divergence, chromosomal and bioacoustic evidence available to date, the four species recognized here cannot be diagnosed by any known phenotypic character. This might be considered a reversal for the taxonomy of the genus, though actually the lack of consistent and reliable diagnostic characters is deeply rooted in its taxonomic history as revealed by a careful examination of the literature.

Other three relevant aspects related to the taxonomy and geographic distributions of *Eupsophus* species can be drawn from the literature. First, the descriptions and diagnoses are difficult to be compared because they include different numbers and types of characters. Second, phenotypic variation is pervasive in the genus, as we show in this study with several examples, but it has been poorly described and practically neglected in the taxonomic research. Third, it has been assumed that the distributions of species of the *roseus* group are mostly allopatric, but the cumulative information of reported localities shows a high degree of overlap and syntopy among these species, most likely a consequence of field misidentifications. We are aware that our proposal does not fully resolve these problems, particularly that of the field identification; however, we do provide a robust phylogenetic backbone to which new populations can be incorporated as sampling gaps are filled. The alternative, maintaining the current taxonomy, it means accepting species separated by very low genetic divergences (*E*. *migueli* and *E*. *altor*) or that are not reciprocally monophyletic (*E*. *contulmoensis* and *E*. *nahuelbutensis*) with no obvious or consistent phenotypic, karyotype or bioacoustic differences.

Biogeographically, the new taxonomic proposal supports a mainly allopatric pattern for the species of the *roseus* group, which concurs with some previous claims [21], but not with the cumulative geographic information of the literature. Our increase of samples from the type localities of nominal species and the addition of geographically intermediate localities suggest that syntopy among species of the *roseus* group is not common and that they, in general, occupy separated areas. In fact, only two possible zones of sympatry were detected by the phylogenetic analysis, located approximately between 39°40’ and 40°S (S4 File). These zones can be included in a narrow strip of the Coastal Range between 38 and 41°S, where the six species of the genus recognized here are present (including the previously insular endemic *E*. *insularis* and the two ones of the *vertebralis* group). On the other hand, the diversity of species along the Andean foothills is maintained despite the taxonomic changes, since only *E*. *roseus* and *E*. *calcaratus* (*roseus* group), and *E*. *vertebralis* and *E*. *emiliopugini* (*vertebralis* group) are found there. Thus, the species richness and distribution ranges remain consistent with an origin of *Eupsophus* on the Coastal Range of southern Chile [18] and suggest that this zone could have served as a glacial refuge [27] for several species of the genus.

The new taxonomy also has important consequences for the conservation of species of the *roseus* group (species of the *vertebralis* group are not under any threatened category [38]), because it implies significant changes in their geographic distributions. According to the IUCN [38], three species (*E*. *contulmoensis*, *E*. *migueli* and *E*. *nahuelbutensis*) are Endangered and one Critically Endangered (*E*. *insularis*), mainly due to their reduced distribution ranges (criterion B), and one is Data Deficient (*E*. *septentrionalis*), although in this last case its synomym *E*. *queulensis* is also listed (Vulnerable by criterion D). A similar classification has been adopted by the Chilean Government through its official instrument “Reglamento de Clasificación de Especies Silvestres” (reviewed in [70]), where the same five species are Endangered and *E*. *roseus* is considered Vulnerable. The new taxonomic scheme implies that *E*. *altor*, *E*. *contulmoensis*, *E*. *nahuelbutensis* and *E*. *septentrionalis* are no longer recognized, the distribution range of *E*. *migueli* is extended to the south (encompassing that of *E*. *altor*), *E*. *insularis* is reported for the first time for the continental area at two localities, and the distribution of *E*. *roseus* is extended almost 100 km to the north by the Coastal Range to include the populations previously assigned to *E*. *septentrionalis*. Although in these three cases there was an increase of the extent of occurrence, the distribution ranges of *E*. *insularis* and *E*. *migueli* still are relative small. Future reassessments should also take into account the ongoing decline of their habitat, the temperate forests. These forests originally covered both the Coastal Range in Chile (from 35 to 43°S approximately) and the Andean foothills in Chile and Argentina, but have been progressively replaced by plantations of exotic trees and currently are severely fragmented, particularly in the Coastal Range [71–73]. Moreover, nothing is known about the population sizes, demographic trends or population ecology of *Eupsophus*, so there are no additional elements outside the geographic distributions and perceived declines or threats, to reassess the conservation categories. Here lies the importance of obtaining a more robust taxonomic and geographic delimitation framework that better reflects the evolutionary history and biogeography of the genus, as a basis for conservation reassessments.

The widespread problem of field identification of *Eupsophus* is certainly due to the high levels of variation in external characters, a phenomenon that was described early in the literature [74, 75] and in the type series of several species (e.g. [67, 69, 76]), but that was subsequently neglected in the taxonomic research. Variation in coloration patterns has been widely recognized in amphibians [77, 78], and has been described in detail in a few species of Chilean amphibians [79, 80]. In the case of *Eupsophus* of the *roseus* group, members of the same population may display distinctive features such as a vertebral line, barred legs, melanic dots on the posterior dorsal region and/or a spot like hourglass on the dorsum, over a uniform or spotted background coloration that can be reddish, orange, yellow, green, gray, brown or even blackish in different shades and combinations (see Fig 1 and S1 and S2 Figs). These potentially infinite combinations of colors and patterns are comparable to the intricate mix of colors and shapes formed by fallen leaves on the forest floor (S1A Fig), where these organisms are usually found, so the high level of polymorphism in dorsal coloration might constitute a camouflage mechanism. In *Rhinoderma darwinii*, other polymorphic frog from the temperate forests that lives in sympatry with *Eupsophus*, an association between body and substrate coloration has been described, which suggests a strategy of crypsis to reduce predation risk [80]. Regarding *Eupsophus* populations, Cei [74] indicated that characteristic coloration patterns could predominate in certain geographic areas, but currently there are no comprehensive descriptive studies of the distribution of color patterns considering a more recent taxonomy. These predominant coloration patterns might be behind the descriptions of some species like *E*. *migueli*, *E*. *contulmoensis* and *E*. *nahuelbutensis*, since they would have their respective “typical” coloration (e.g. [16, 21]). Likewise, the other characters more frequently used in the diagnoses, iris coloration, snout profile and shape of the xiphisternum, could exhibit certain levels of geographic variation, but our observations and the available evidence suggest that any apparent consistent difference among populations is blurred when a higher number of specimens is analyzed (e.g. [36]).

The claims suggesting that the richness of anuran species of the temperate forests from southern Chile has been underestimated due to cryptic diversity [34, 35] are mainly based on the discovery of *E*. *altor*, which adds to a series of congeneric species with restricted range. Nevertheless, are the known patterns of phenotypic variation of *Eupsophus* consistent with a cryptic diversity phenomenon? We provide two arguments that challenge this statement. First, by definition, cryptic species are morphologically indistinguishable so they are hypothesized when high levels of intraspecific genetic divergence are detected [81]. Instead, we show that the genetic divergences between the nominal species *E*. *contulmoensis* and *E*. *nahuelbutensis*, and between *E*. *migueli* and *E*. *altor* are extremely low, and when novel geographically intermediate lineages are considered, the divergences among some nominal species decrease (e.g. *E*. *roseus* and *E*. *septentrionalis*). Without significant genetic divergences among nominal species and novel lineages, there is no primary evidence to suggest the presence of undetected cryptic species. Second, if cryptic species predominate in the *roseus* group, a morphological uniformity among them would be expected. The literature analysis and our observations reveal a rather different pattern since the intrapopulation variation in the three most commonly used (supposedly) diagnostic characters is as high as the interspecific variation. Therefore, populations are not morphologically indistinguishable from each other because they are morphologically uniform but rather because they exhibit so much variation that it is not possible to define fixed characteristics that differentiate them. One explanation for this high level of polymorphism is the widespread occurrence of more than one species in the same place, but our results show that sympatry among highly genetic divergent lineages, irrespective of their taxonomic status, is rare in the *roseus* group.

Integrative approaches, using mainly morphological (and/or morphometric), molecular and bioacoustic evidence, have been increasingly applied to revisit and resolve taxonomic problems in diverse groups of amphibians, revealing in many instances cryptic diversity ([6, 7, 11, 82–84], just to mention a few recent examples), but also frequently discordant patterns of variation among data sets (e.g. [85–89]). These discordances can be ultimately attributed to the high levels of intra and interspecific phenotypic variation, which may largely explain the mismatch between the accepted taxonomy of those groups and the new obtained evidence, but the quoted studies have rarely addressed those discrepancies taking into account a thorough literature review [11, 89]. In the case of *Eupsophus*, the detailed examination of more than thirty-five years of taxonomic work revealed not only discrepancies among the morphological, chromosomal, bioacoustics and genetic evidence, but also a low level of variation of karyotypes and advertisement calls in the *roseus* group. These last two lines of evidence, used regularly in the anuran taxonomic research (particularly the calls, see examples above), suggest that the species diversity in this group has been overestimated. Therefore, considering the patterns of intra and interpopulation variation in morphology, we argue that local, non-fixed phenotypic differences were overemphasized in the descriptions of some species of the *roseus* group, which resulted in unhelpful diagnoses that finally led to confusion to define their taxonomic and geographic boundaries. In this context, our conservative species diversity estimation constitutes a starting point to understand the evolutionary and ecological causes of the extreme phenotypic variation of *Eupsophus*.

## Supporting information

S1 File Taxonomic overview (including Table A). (DOCX)

S2 File Bioacoustic studies (including Table B). (DOCX)

S3 File Chromosome studies. (DOCX)

S4 File Geographic distribution overview of the *Eupsophus roseus* group. (DOCX)

S5 File Compilation of localities of the genus *Eupsophus* (including Fig A). (DOCX)

S1 Fig Examples of intrapopulation variation of diagnostic characters frequently used in the taxonomy of the *Eupsophus roseus* group. (DOCX)

S2 Fig Additional examples of phenotypic external variation in two new populations of *Eupsophus roseus*. (DOCX)

S3 Fig Graphical results of the analysis ABGD. (DOCX)

S1 Table Kimura two-parameter distances among the seven candidate species (CSs) obtained with mPTP and relaxed ABGD analyses. (DOCX)

S2 Table Biological material used to obtain DNA sequences in this study and GenBank accession numbers. (DOCX)
