# Supplementary material for: Species delimitation in frogs from South American temperate forests: The case of Eupsophus, a taxonomically complex genus with high phenotypic variation
Source: PLoS One. 2017 Aug 15;12(8):e0181026. doi: 10.1371/journal.pone.0181026 (PMC5557580; doi:10.1371/journal.pone.0181026)
Supplement: S1 File — (DOCX) [file pone.0181026.s001.docx]

**S1 File: Taxonomic overview**

Current content of the genus

The current delimitation of the genus was achieved in the late seventies [37, 76, 90], when only three species were recognized (*E. roseus* described by Duméril & Bibron [91] as *Cystignathus roseus*, *E. vertebralis* by Grandison [92], and *E. migueli* by Formas [76]). Since that time there was a continuous description of species (*E. contulmoensis* [33], *E. emiliopugini* [93], *E. nahuelbutensis* [67], *E. septentrionalis* [68], *E. queulensis* [69] and *E. altor* [34]) or revalidations in the case of *E. calcaratus* and *E. insularis* [41] (described as *Cacotus calcaratus* by Günther [94] and *Cystignathus* (*Borborocoetus*) *insularis* by Philippi [95], respectively). Later, *Eupsophus queulensis* was synonymized with *E. septentrionalis* by Blotto et al. [18], so the number of species was reduced to ten. The division of these species into two groups, *roseus* (*E. roseus*, *E. calcaratus*, *E. insularis*, *E. migueli*, *E. contulmoensis*, *E. nahuelbutensis*, *E. septentrionalis* and *E. altor*) and *vertebralis* (*E. vertebralis* and *E. emiliopugini*), was first proposed by Formas et al. [24] based on chromosome evidence.

Diagnostic characters of *Eupsophus* species

We focus only on diagnostic characters because they are the basis for distinguishing one species from the others [96]. In Table A we summarize the diagnostic characters of nine species of the *roseus* group, including *E. queulensis*. In the case of *E. roseus*, *E. calcaratus* and *E. insularis*, those characters were obtained from additional sources because the original descriptions are very brief and the latter two species were redescribed by Formas & Vera [41]. Unlike all other species, the diagnosis of *E. altor* includes a call parameter and reproductive traits (see main text), and only in the case of this species and *E. septentrionalis*, molecular characters were included. The characters used more frequently in the diagnoses of the species of the *roseus* group are the general coloration, the color of the iris, the shape of the snout, the shape of the xiphisternum (the cartilaginous plate at the lowermost end of the sternum) and, to a lesser extent, the karyotype (Table A). All these morphological characters show high levels of intraspecific variation, even in the type series (body coloration in *E. insularis*, *E. migueli*, *E. contulmoensis*, *E. nahuelbutensis*, *E. septentrionalis* and *E. queulensis*; snout profile in *E. nahuelbutensis*; Table A). Variation in dorsal and/or ventral coloration patterns is pervasive in the genus, as exemplified by specimens from the type localities of most species (Fig 1 and S1 Fig) and from the new localities (S2 Fig). A conspicuous polymorphic characteristic (present, absent or variable in length and width; more typical of the *vertebralis* group [92, 93]) is a mid-dorsal (vertebral) line of whitish or yellowish color in almost all species (Fig 1 and S1 and S2 Figs). Iris coloration seems to be a more constant character since there are no reports of variation in the species descriptions, but it is difficult to distinguish the differences in this trait among some species (bronze-yellow, yellowish, light yellow and yellow, Table A); in contrast, *E. roseus* have the iris orange [41]. Nuñez [21] provided partially different descriptions, suggesting that there is intraspecific variation in this trait: the iris color of *E. calcaratus* and *E. nahuelbutensis* is “generally” yellow, whereas that of *E. roseus*, *E. migueli* and *E. contulmoensis* “can be” orange. S1 and S2 Figs show several examples of intrapopulation variation in iris coloration. The snout profile, both in dorsal and lateral view, is another highly variable character within populations from both type and new localities (S1 and S2 Figs). Finally, the shape of the xiphisternum has been the osteological character most frequently used in the descriptions and diagnoses, where implicitly was considered as fixed. However, Díaz [36] examined the form of the xiphisternum in samples from the type localities of *E. roseus* (Valdivia, n = 37) and of *E. migueli* (Mehuín, n = 45), finding four types of xiphisternum (rounded, pointed, notched and seminotched) in *E. migueli* and three in *E. roseus* (notched condition was not found). In both species, the rounded xiphisternum was the most frequent condition. Note that a notched xiphisternum is considered as diagnostic of *E. migueli* (Table A).

Table A. Phenotypic, karyotypic and genetic characters included in the diagnoses of nine nominal species of the *roseus* group. Species are ordered by date of description (references that include the respective descriptions and diagnoses are indicated next to the name). *Eupsophus queulensis* (synonym of *E. septentrionalis* [18]) was included to emphasize the heterogeneity of characters used in the diagnoses. The original descriptions and diagnoses of *E. roseus*, *E. calcaratus* and *E. insularis* are very brief and were done under generic names that currently are not valid, so we compiled their characters from additional sources [21, 41]. The character states are described as they appear in the original descriptions, and in most cases, they were transcribed literally. Note that new kind of characters have continuously been added over time, but not necessarily implying an increase in their total number. Blank cells indicate that character was not included in the diagnosis of that species. The V in parentheses indicates that the character was variable in the type series.

| **character** | ***E. roseus*** [91, 21, 41] | ***E. calcaratus*** [94, 41] | ***E. insularis*** [95, 41] | ***E. migueli*** [76] | ***E. contulmoensis*** [33] | ***E. nahuelbutensis*** [67] | ***E. septentrionalis*** [68] | ***E. queulensis*** [69] | ***E. altor*** [34] |
| --- | --- | --- | --- | --- | --- | --- | --- | --- | --- |
| **Body coloration** | body with brown tones on a pink background; ventral area transparent or whitish |  | dark brown with irregular yellow spots on the dorsum and legs (V) | dark venter and white brilliant irregular spots (V) | dark purple dorsal pigmentation and bright yellow belly (V) | white belly with dark brown marmorino (V) | dark brown to blackish on a light gray to leaden background (V) | two melanic dots on dorsal region and reddish ventral surfaces (V) |  |
| **Upper part of iris** | orange | bronze-yellow |  | bronze-yellow | bronze-yellow |  | light yellow | yellow |  |
| **Shape of snout** |  | pointed in  dorsal and lateral view, noticeably protruding over the lower jaw^a^ |  |  |  | sloping in lateral view (V) |  | truncate in lateral view |  |
| **Xiphisternum** |  |  | truncated and slightly notched | notched^b^ |  | ample in its middle portion and rounded at its extreme |  | without a notch |  |
| **Canthus rostralis** | thin and short |  |  |  |  | ample and extended |  |  |  |
| **Cloacal fold** |  |  |  |  |  | marked |  |  |  |
| **Epicoracoids** | right over left |  |  |  |  |  |  | left epicoracoid superimposed to the right one |  |
| **Carpal tubercles** |  |  |  |  | inner palmar tubercle prominent |  |  | prominent external and internal tubercles |  |
| **Tips of toes** |  | rounded and prominent |  |  |  |  |  |  |  |
| **Other osteological or morphological characters** | vomerine teeth arranged in a transverse row | cutaneous spur (calcar) at the heels^c^; prevomers in narrow contact | prevomerine teeth below the choanae |  |  |  | skull morphology |  |  |
| **Karyotype** | eight pairs of biarmed chromosomes |  |  | 16 acrocentric chromosomes |  |  |  | heteromorphic sexual chromosomes, and secondary constriction at the fourth pair |  |
| **Allozymes** |  |  |  |  |  |  | allozyme pattern (V) |  |  |
| **Reproductive traits** |  |  |  |  |  |  |  |  | early winter breeding season and terrestrial tadpoles |
| **Advertisement call** |  |  |  |  |  |  |  |  | spectral elements reaching 20 kHz |
| **Genetic divergence** |  |  |  |  |  |  |  |  | nine nucleotide site substitutions in the mitochondrial control region from *E. migueli* |

^a^Formas & Vera [41] used this character to differentiate *E. calcaratus* from *E. roseus*, but they did not describe the snout profile of *E. roseus* (they only show a drawing of the head in lateral profile).

^b^Díaz [36] described four types of xiphisternum in the type locality of this species and three in that of *E. roseus* (see details in the text).

^c^This structure is considered characteristic of this species (e.g. [16, 31, 94]), but has not been included in its diagnosis [41]; Nuñez et al. [31] mentioned that this character is also present in some specimens of *E. roseus*.

**References (not cited in the main text)**

1. Formas JR. The identity of the frog *Eupsophus vanzolinii* from Ramadillas, Nahuelbuta Range, Southern Chile. Proc Biol Soc Wash. 1980;93: 920-927.
2. Duméril AMC, Bibron G. Erpétologie genérale ou histoire naturelle complète des reptiles. Paris: Librarie Enclyclopedique de Roret; 1841.
3. Grandison AGC. Chilean species of the genus *Eupsophus* (Anura: Leptodactylidae). Bull Br Mus Nat Hist Zool. 1961;8: 111-149.
4. Formas JR. A new species of *Eupsophus* (Amphibia: Anura: Leptodactylidae) from Southern Chile. Proc Biol Soc Wash. 1989;102: 568-576.
5. Günther A. Account of the zoological collections made collected during the survey of H.M.S. “Alert” in the Straits of Magellan and on the coast of Patagonia. III. Reptiles, batrachians, and fishes. Proc Zool Soc Lond. 1881; 18-22.
6. Philippi RA. Suplemento a los batraquios chilenos descritos en la Historia Física i Política de Chile de don Claudio Gay. Santiago de Chile: Librería Alemana de José Ivens; 1902.
7. Winston JE. Describing species: practical taxonomic procedure for biologists. New York: Columbia University Press; 1999.
