# Supplementary material for: Species delimitation in frogs from South American temperate forests: The case of Eupsophus, a taxonomically complex genus with high phenotypic variation
Source: PLoS One. 2017 Aug 15;12(8):e0181026. doi: 10.1371/journal.pone.0181026 (PMC5557580; doi:10.1371/journal.pone.0181026)
Supplement: S2 File — (DOCX) [file pone.0181026.s002.docx]

**S2 File: Bioacoustic studies**

Vocalizations have been described in nine species of the genus (excepting *E. nahuelbutensis*) ([22, 23, 34, 40, 93, 97, 98]; see details below in Table B). Comparisons of advertisement calls have revealed contrasting patterns of variation within the species groups: these calls are different between the two species of the *vertebralis* group (number and duration of notes [93]), but are remarkably similar within the *roseus* group [22, 23, 34, 97, 98]. In fact, all parameters used to characterize calls exhibit a high degree of overlap so they are not useful in differentiating the species of the *roseus* group (Table B). Recently, a quantitative measure of the advertisement call, the frequency of spectral elements, was included in the diagnosis of *E. altor* ([34]; see comment in the section Synonimization of *E. altor*, in the main text).

Table B. Summary of advertisement call parameters of *Eupsophus*. Species are ordered by group (*roseus* and *vertebralis*) and then by year of description and locality. Mean and/or range (in brackets) of each parameter (N/C: notes per call; CL: call length; FF: fundamental frequency; DF: dominant frequency) are given, except for the maximum frequency (MF), which was reported by Nuñez et al. [34] as a limit.

| **species** | **source** | **locality** | **N/C** | **CL (ms)** | **FF (Hz)** | **DF (Hz)** | **MF (kHz)** |
| --- | --- | --- | --- | --- | --- | --- | --- |
| ***E. roseus*** | [40] | Huachocopihue | 1 | 200 (190-210) | - | 2200 (1600-2900) | - |
| ***E. roseus*** | [99] | Lago Tinquilco | 1 | 158 (124-235) | 633 (346-1019) | 1871 (1503-2167) | - |
| ***E. roseus*** | [97] | Valdivia | 1 | 105 (70-160) | - | 1291 (1250-1350) | - |
| ***E. calcaratus*** | [22] | Puntra | 1 | 190 (150-210) | - | 1100-2700 | - |
| ***E. calcaratus*** | [99] | La Picada | 1 | 192 (112-262) | 776 (447-1104) | 2157 (1805-2407) | - |
| ***E. insularis*** | [23] | Isla Mocha | 1 | 160 (140-180) | - | 1500-2100 | - |
| ***E. migueli*** | [22] | Mehuín | 1 | 240 (200-350) | 450 (390-987) | 1835 (1500-2500) | - |
| ***E. migueli*** | [97] | Mehuín | 1 | 208 (160-260) | - | 1633 (1170-1820) | - |
| ***E. contulmoensis*** | [23] | M.N. Contulmo | 1 | 180 (150-200) | - | 1100-2000 | - |
| ***E. septentrionalis*^a^** | [98] | R.N. Los Queules | 1 | 135 (46-182) | - | 1818 (1464-2326) | - |
| ***E. altor*** | [34] | Parque Oncol | 1 | 336 (290-360) | 756 (304-1298) | 1882 (1317-2098) | 20^b^ |
| ***E. vertebralis*^c^** | [40] | Mehuín | 5 (4-6) | 600 (400-800) | - | 1900 (1100-2500) | - |
| ***E. vertebralis*** | [93] | Mehuín | 5 (4-6) | 89 (62-187) | - | 1154 (600-1680) | - |
| ***E. vertebralis*** | [97] | Mehuín | 5.6 (3-8) | 641 (400-880) | - | 932 (700-1110) | - |
| ***E. vertebralis*** | [21] | not specified | 4-6 | 600 (400-800) | - | 700-1110 | - |
| ***E. emiliopugini*** | [93] | Puntra | 2 | 203 (132-250) | 85-633 | 1132 (500-2000) | - |
| ***E. emiliopugini*** | [100] | La Picada | 1?^d^ | 255 (181-314) | - | 1062 (636-1459) | - |
| ***E. emiliopugini*** | [21] | not specified | 2 | 640 (400-880) | - | 507-1320 | - |
| ***E. emiliopugini*** | [101] | La Picada | 1-2 | 255 (177-342) | - | 1053 (723-1401) | - |

^a^As *E. queulensis*.

^b^Nuñez et al. [34] only show the sonogram for *E. altor*, but mention as personal observation that *E. roseus* and *E. migueli* do not reach 15 kHz.

^c^As *E. vittatus*.

^d^The number of notes is not explicitly indicated.

**References (not cited in the main text)**

1. Formas JR. A new species of *Eupsophus* (Amphibia: Anura: Leptodactylidae) from Southern Chile. Proc Biol Soc Wash. 1989;102: 568-576.
2. Penna M, Veloso A. Vocal diversity in frogs of the South American temperate forest. J Herpetol. 1990;24: 23-33.
3. Opazo D, Velásquez N, Veloso A, Penna M. Frequency-Modulated vocalizations of *Eupsophus queulensis* (Anura, Cycloramphidae). J Herpetol. 2009;43: 657-664.
4. Márquez R, Penna M, Marques P, Do Amaral JPS. Diverse types of advertisement calls in the frogs *Eupsophus calcaratus* and *E. roseus* (Leptodactylidae): a quantitative comparison. Herpetol J. 2005;15: 257-263.
5. Penna M, Solís R. Extent and variation of sound enhancement inside burrows of the frog *Eupsophus emiliopugini* (Leptodactylidae). Behav Ecol Sociobiol. 1999;47: 94-103.
6. Penna M, Narins PM, Feng AS. Thresholds for evoked vocal responses of *Eupsophus emiliopugini* (Amphibia, Leptodactylidae). Herpetologica. 2005;61: 1-8.
