# Supplementary material for: Species delimitation in frogs from South American temperate forests: The case of Eupsophus, a taxonomically complex genus with high phenotypic variation
Source: PLoS One. 2017 Aug 15;12(8):e0181026. doi: 10.1371/journal.pone.0181026 (PMC5557580; doi:10.1371/journal.pone.0181026)
Supplement: S3 File — (DOCX) [file pone.0181026.s003.docx]

**S3 File: Chromosome studies**

Chromosome evidence in *Eupsophus* has been used mainly to establish differences among species, assuming implicitly or explicitly [102] that amphibian species have a characteristic karyotype. The karyotypes of nine species have been described, excepting *E. nahuelbutensis*, which according to Nuñez [21] has 30 chromosomes. So, all species of the *roseus* group have 30 chromosomes, whereas those of *vertebralis* group have 28 (summarized in [69]). Three of these species have heteromorphic sex chromosomes, *E. migueli* [103], *E. insularis* [104] and *E. septentrionalis* (as *E.* *queulensis* [69]), where the pair 14 is telocentric in females and is heteromorphic (one telocentric and one metacentric chromosome) in males (which implies different fundamental numbers (FN) for each sex, 44 and 45, respectively). Intrapopulation variation in karyotypes only is inferred when comparing different studies on the same population. For example, chromosome morphology and position of the secondary constriction vary among studies in *E. migueli* [69, 76, 102, 103, 105, 106] and in *E. roseus* [76, 102, 106] from their type localities. Also, the comparison of different studies reveals differences in chromosome morphology [24, 105] and the presence [105] or absence [24, 69] of a secondary constriction in *E. vertebralis* from Mehuín. Likewise, the heteromorphic sex chromosomes of *E. migueli* from Mehuín, first described by Iturra & Veloso [103], were not observed in previous studies of the species [76, 102, 105]. All these examples imply different karyotypes for the same species, but without describing intraspecific or intrapopulation variation in the same study (despite that in some studies more than ten samples of both sexes were used), which suggests differences dependent on the observer. In summary, all species of the *roseus* group have the same chromosome number and one secondary constriction, but the only consistent difference among species is the presence (*E. migueli*, *E. insularis* and *E. septentrionalis*; presumably *E. altor*, because females have NF = 44 [34] like *E. migueli* and *E. insularis*) or absence of heteromorphic sex chromosomes (*E. roseus*, *E. calcaratus* and *E. contulmoensis*). *Eupsophus nahuelbutensis* presumably belongs to this last group because is phylogenetically related to *E. contulmoensis* and *E. roseus* [18].

**References (not cited in the main text)**

1. Formas JR. Systematic problems in the frog species *Eupsophus roseus* (Anura: Leptodactylidae) detected by karyological analysis. Experientia. 1978;34: 446.
2. Iturra P, Veloso A. Evidence for heteromorphic sex chromosomes in male amphibians (Anura: Leptodactylidae). Cytogenet Cell Genet. 1981;31: 108-110.
3. Cuevas CC, Formas JR. Heteromorphic sex chromosomes in *Eupsophus insularis* (Amphibia: Anura: Leptodactylidae). Chromosome Res. 1996;4: 467­470.
4. Bogart JP. Systematics problems in the amphibian family Leptodactylidae (Anura) as indicated by karyotypic analysis. Cytogenetics. 1970;9: 369­383.
5. Iturra P, Veloso A. Further evidence for early sex chromosome differentiation of Anuran species. Genetica. 1989;78: 25-31.
