# Supplementary material for: Species delimitation in frogs from South American temperate forests: The case of Eupsophus, a taxonomically complex genus with high phenotypic variation
Source: PLoS One. 2017 Aug 15;12(8):e0181026. doi: 10.1371/journal.pone.0181026 (PMC5557580; doi:10.1371/journal.pone.0181026)
Supplement: S4 File — (DOCX) [file pone.0181026.s004.docx]

**S4 File:** **Geographic distribution overview of the *Eupsophus* *roseus* group**

General patterns and sources of maps

The genus *Eupsophus* distributes in the temperate forests of Chile and Argentina, between 35°50’ and 49°25’S approximately [16, 45, 68], although in Argentina its distribution is more restricted (39°20’-43°S, [107, 108]). The two species groups have widely overlapping distribution ranges in Chile and Argentina [21], but the *roseus* group has the same distribution as the entire genus, surpassing the *vertebralis* group towards the north (about 150 km) and to the south (about 400 km) (Fig 2). In the literature is broadly held that species within both groups have allopatric distributions [21, 22, 31, 93], but there are three reported cases of syntopy, in the same publication, among species of the *roseus* group in Chile (Fig 2 and Fig A in S5 File): *E. roseus* and *E. migueli* in Mehuín ([32], type locality of *E. migueli*; coordinates in Table 1), *E. roseus* and *E. contulmoensis* in M.N. Contulmo ([33], type locality of *E. contulmoensis*), and *E. nahuelbutensis* and *E. calcaratus* in P.N. Nahuelbuta ([67], type locality of *E. nahuelbutensis*). Additional instances of syntopy can be inferred by combining different sources: *E. roseus*, *E. calcaratus* and *E. nahuelbutensis* in P.N. Nahuelbuta [31, 67], *E. roseus* and *E. migueli* in Los Molinos [40, 76] and in Queule [43], *E. roseus* and *E. altor* in Alepúe [18, 34], and *E. contulmoensis* and *E. nahuelbutensis* in Ramadillas [21, 44] (Figs 2 and A). In addition, the geographic delimitation between *E. calcaratus* and *E. roseus*, the two species with the widest distribution ranges within the *roseus* group, is not well defined in Chile and Argentina [18, 31]. The limit between both species in Chile would be the Calle-Calle river basin, around 39°50’S [31], but there are records of both that surpass that limit (Fig A). Nevertheless, the most important biogeographic problem within the genus it is to define the distribution of *E. roseus* with respect to other species of the *roseus* group. Four species (*E. migueli*, *E. contulmoensis*, *E. nahuelbutensis* and *E. altor*) have been described within the range limits of *E. roseus* (between Concepción and Valdivia, 36°50’-39°48’S, according to Formas et al. [30]), but none of those studies clarified the levels of sympatry or syntopy among those species. This issue is further complicated for the numerous cases of reported or inferred syntopy among species mentioned above.

The last compilation of localities of *Eupsophus* was done by Nuñez [21], who included the eight species described at that date (*E. roseus*, *E. calcaratus*, *E. insularis*, *E. vertebralis*, *E. migueli*, *E. contulmoensis*, *E. emiliopugini* and *E. nahuelbutensis*). Later, Rabanal & Nuñez [16] provided distribution maps for the same eight species (depicted as areas), but the most recent maps (extent of the occurrence of ten species, including *E. septentrionalis* and *E. queulensis*, but not *E. altor*) are found in the IUCN Red List of Threatened Species^TM^ [38]. Although the assessments of the IUCN date from 2008, the maps do not reflect exactly the historical records of the species, particularly of *E. roseus*. Following, we detail the sources of the most relevant localities showed in the maps of Fig A, highlighting the conflictive information and discrepancies among the geographic data of species of the *roseus* group. All localities mentioned in the following sections are included in the list of the S5 File and in Fig A.

*Eupsophus roseus*

The distribution of this species is the more difficult to define from the literature because its limits, particularly the northern, differ among sources and four species have been described within its minimally accepted range in Chile. For example, Formas [14] and Formas et al. [30] indicated that the species reaches the city of Concepción by the north (36°50’S), while later sources limit it to the Nahuelbuta Range (37°50’S approximately [16, 28 in 38, 31]) or further south (P.N. Tolhuaca, 38°13’S [21]). However, two records (Tomé [74, 75], as *E. grayi*, and Tumbes [92]), ignored in the posterior literature, implied that *E. roseus* is present north of Concepción. Its southern limit in Chile would be the Calle-Calle river basin (around 39°50’S), from where is replaced by *E. calcaratus* southwards according to Nuñez et al. [31], but there are records of both species that surpass that limit. Thus, *E. roseus* has been reported further south in Camino Viejo a La Unión [40] and in Reserva Costera Valdiviana [109], and conversely, *E. calcaratus* further north in P.N. Nahuelbuta (type locality of *E. nahuelbutensis* [67], where also *E. roseus* would be present [31]) and in Mississipi [43] (Fig A). Therefore, considering the extreme localities reported in the literature, *E. roseus* distributes along the coastal area between 36°35’ and 40°S, encompassing completely the distribution range of four species of the *roseus* group described later (see below), and is sympatric with *E. calcaratus* in a narrow zone of its southern limit. On the occidental Andean foothills, *E. roseus* has been reported between P.N. Tolhuaca ([21], although Blotto et al. [18] suggested that this population would represent an undescribed species) and Huilo Huilo (39°51’S [30]). The presence of *E. roseus* in Argentina has been debated (e.g. [108]), where some populations have been unsteadily assigned to *E. roseus* and/or to *E. calcaratus* (discussed in [18]), but the results of Blotto et al. [18] and ours confirm its presence in that country. However, the two samples of Argentina identified as *E. roseus* by Blotto et al. [18] would be flanked to the south and north by populations of *E. calcaratus*, if all the historical localities assigned to the latter species are considered [107].

Sympatric species with *E. roseus*

The four species described within the distribution limits of *E. roseus* are: *E. migueli* [76], *E. contulmoensis* [33], *E. nahuelbutensis* [67] and *E. altor* [34]. *Eupsophus migueli* was described from Mehuín (type locality) and Los Molinos [76], near Valdivia city (type locality of *E. roseus* [110]). Some previous studies included specimens from Mehuín as *E. roseus* [102, 105], but there were reports of *E. roseus* after the description of *E. migueli* in Mehuín [32, 43] and Los Molinos [40]. Later, the distribution of *E. migueli* was extended to San José de la Mariquina [43], Colehual Alto [34] and Queule ([18], where *E. roseus* also has been recorded [43]), but all these localities are situated in a narrow coastal strip between 39°22’ and 39°51’S (map B of Fig A). *Eupsophus contulmoensis* was known initially only from its type locality, M.N. Contulmo (e.g. [23, 21, 68, 111]), but Ortiz & Ibarra-Vidal [44] pointed its presence further north to Ramadillas (37°20’S), and indicated that is widely distributed in the maritime slopes of the Nahuelbuta Range from south of Biobío river (north of Ramadillas) to the latitude of Tirúa (38°20’S). The other endemic from Nahuelbuta Range is *E. nahuelbutensis*, which is known from P.N. Nahuelbuta (its type locality, e.g. [21, 112, 113]), Ramadillas (e.g. [112]) and Rucapehuén (e.g. [21]). According to these records, *E. contulmoensis* and *E. nahuelbutensis* are syntopic in Ramadillas, but *E. nahuelbutensis* also occurs in syntopy with *E. roseus* (see above) and even *E. calcaratus* [67] in P.N. Nahuelbuta (see below) (Fig 2A). *Eupsophus altor* was reported originally from four localities [34], but a map by Nuñez et al. [35] shows six points without mentioning the localities. All these localities are found in a narrow strip of the Chilean Coastal Range between 39°29’ and 39°42’S, so according to the literature records, *E. altor* would be completely surrounded by populations of *E. migueli* and *E. roseus* (Fig 2B). In one of the original localities of *E. altor*, Alepúe [34], the presence of *E. roseus* [18] also has been recorded, which implies the sympatry of both species in the northern end of the distribution of *E. altor* (around 39°29’S, locality 43 of Fig A).

Other species of the *roseus* group

*Eusophus calcaratus* has the widest range within the genus (Fig 2). Formas & Vera [41], who revalidated this species, analyzed specimens from Cordillera Pelada (40°10’S) to Chiloé Island, its imprecise type locality (several localities have been reported in this island, between 41°50’ and 43°06’S, Fig A). Later, Nuñez et al. [31] and Nuñez [21] reviewed its distribution expanding it northward to Tres Chiflones (40°02’S) and southward to Puerto Edén (49°08’S). Nuñez et al. [31] defined the boundary between *E. roseus* and *E. calcaratus* on the Calle-Calle River basin, around 39°50’S. However, as mentioned previously, two records of *E. calcaratus* surpass this limit northward: P.N. Nahuelbuta (Nuñez [21] dismissed this record because he could not confirm its presence there) and Mississipi. At the other end, Asencio et al. [45] reported various localities around and south of Puerto Edén, until Bahía Broome (~49°25’S), which constitutes the known southern limit of the genus. *Eupsophus insularis*, recognized as a full species by Formas & Vera [41], until now was known only at Mocha Island, but here we discovered it in two continental localities, Primer Agua and Camino a Villa Las Araucarias (Fig 3). Coastal Range populations previously assigned to *E. septentrionalis* and its synonym *E. queulensis* (around R.N. Los Ruiles, R.N. Los Queules, and Trehuaco [68, 69]; Fig A) were described beyond of the boundaries of *E. roseus* and currently constitute the northern limit of the genus (35°50’S). Both species were syntopic at R.N. Los Queules (35°59’S), but the maps of the IUCN [38] show distribution areas north of 35°50’S for both.

**References (not cited in the main text)**

1. Grandison AGC. Chilean species of the genus *Eupsophus* (Anura: Leptodactylidae). Bull Br Mus Nat Hist Zool. 1961;8: 111-149.
2. Formas JR. A new species of *Eupsophus* (Amphibia: Anura: Leptodactylidae) from Southern Chile. Proc Biol Soc Wash. 1989;102: 568-576.
3. Formas JR. Systematic problems in the frog species *Eupsophus roseus* (Anura: Leptodactylidae) detected by karyological analysis. Experientia. 1978;34: 446.
4. Bogart JP. Systematics problems in the amphibian family Leptodactylidae (Anura) as indicated by karyotypic analysis. Cytogenetics. 1970;9: 369­383.
5. Ubeda CA. *Eupsophus calcaratus* (Anura, Leptodactilydae): ampliación de su distribución geográfica y hábitats en Argentina. Cuad Herpetol. 2000;14: 71-74.
6. Vaira M, Akmentins M, Attademo M, Baldo D, Barrasso D, Barrionuevo S, et al. Categorización del estado de conservación de los anfibios de la República Argentina. Cuad Herpetol. 2012;26: 131-159.
7. Cárdenas DR, Veloso A, de Sá RO. The tadpole of *Eupsophus queulensis* (Anura, Cycloramphidae). Alytes. 2007;25: 45-54.
8. Ortiz JC, Lescure J. Les types d’amphibiens anoures du Chili dans les collections du Muséum national d’histoire naturelle, Catalogue critique. Bull Mus Natn Hist Nat, Paris. 1989;11: 113-122.
9. Formas JR, Lacrampe S, Brieva L. Allozymic and morphological differentiation among three South American frogs, genus *Eupsophus* (*E. roseus*, *E. insularis* and *E. contulmoensis*). Comp Biochem Physiol B. 1992;102: 57-60.
10. Úbeda CA, Nuñez JJ. New parental care behaviours in two telmatobiine genera from temperate Patagonian forests: *Batrachyla* and *Eupsophus* (Anura: Leptodactylidae). Amphibia-Reptilia. 2006;27: 441-444.
11. Nuñez JJ, Úbeda CA. The tadpole of *Eupsophus nahuelbutensis* (Anura: Neobatrachia): external morphology, chondrocranium, and comments on its natural history. Zootaxa. 2009;2126: 58-68.
