# Supplementary material for: Species delimitation in frogs from South American temperate forests: The case of Eupsophus, a taxonomically complex genus with high phenotypic variation
Source: PLoS One. 2017 Aug 15;12(8):e0181026. doi: 10.1371/journal.pone.0181026 (PMC5557580; doi:10.1371/journal.pone.0181026)
Supplement: S5 File — (DOCX) [file pone.0181026.s005.docx]

**S5 File: Compilation of localities of the genus *Eupsophus***

List of localities

This list contains a selection of literature records of the ten species of the genus and the new localities included in the phylogenetic/delimitation analyses (see main text), ordered by species (Fig A). Localities where there would be syntopy among species of the *roseus* group according to the accumulated information of the literature are indicated in bold.

*Eupsophus septentrionalis* (map A): 1) R.N. Los Ruiles, 2) R.N. Los Queules, 4) Trehuaco.

*Eupsophus roseus* (maps A and B): 5) Tomé, 6) Tumbes, 7) Concepción (Cerro Caracol), 16) **P.N. Nahuelbuta**, 17) **M.N. Contulmo**, 22) Primer Agua (*Eupsophus* cf. *roseus*, see Table 1), 23) 10 km west Galvarino, 25) Rucamanque, 26) Cerro Ñielol, 29) Lago Tinquilco, 30) Cuesta Lastarria, 33) Lago Pellaifa, 34) Lago Paimún (Argentina), 35) Termas de Epulafquén (Argentina), 36) Desembocadura del Lago Riñihue, 37) Huilo Huilo, 39) **Queule**, 40) **Mehuín**, 43) **Alepúe**, 46) Iñipulli, 47) Bosque San Martín, 50) Valdivia, 51) Cuesta de Soto, 52) Huachocopihue, 53) **Los Molinos**, 54) Camino Viejo a la Unión, 55) Reserva Costera Valdivia.

*Eupsophus nahuelbutensis* (map A): 12) **Ramadillas**, 13) Rucapehuén, 16) **P.N. Nahuelbuta**.

*Eupsophus contulmoensis* (map A): 12) **Ramadillas**, 17) **M.N. Contulmo**.

*Eupsophus insularis* (map A): 21) Isla Mocha.

*Eupsophus migueli* (map B): 38) Colehual Alto, 39) **Queule**, 40) **Mehuín**, 44) San José de la Mariquina, 53) **Los Molinos**.

*Eupsophus altor* (map B): 43) **Alepúe**, 45) Chanchán, 48) Parque Oncol, 49) Curiñanco.

*Eupsophus calcaratus* (maps A, B, and C): 16) **P.N. Nahuelbuta**, 41) Mississipi, 57) Chaihuín, 58) Tres Chiflones, 59) Lagunas Gemelas, 60) Camino a P.N. Alerce Costero, 61) La Barra, 62) Namun Lahual, 63) Pucatrihue, 64) P.N. Puyehue, 65) Catrihuala, 66) La Picada, 67) Río El Manzano, 68) Llico Bajo, 69) Río Correntoso, 70) Guabún, 71) Caulín, 72) Puntra, 73) Metahue (Isla Butachauques), 74) Chiloé (?, unspecified location), 75) Cucao, 76) Terao, 77) Caleta Tendedor (Isla Talcán), 78) El Amarillo, 79) Yaldad, 80) Río Chico, 81) Villa Santa Lucía, 82) Isla Guafo, 83) Raúl Marín Balmaceda, 84) La Junta, 85) Lago Verde, 86) Queulat, 87) Isla Chaculay, 88) Puerto Aysén, 89) Isla Guerrero, 90) Puente Traihuanca, 91) Canal de Ofqui, 92) Puerto Bertrand, 93) Tortel, 94) Isla Juan Stuven, 95) Lago Quetru, 96) Puerto Edén, 97) Bahía Broome.

*Eupsophus vertebralis* (map D): 12) Ramadillas, 17) M.N. Contulmo, 19) P.N. Tolhuaca, 40) Mehuín, 98) Máfil, 50) Valdivia, 99) Llancahue, 58) Tres Chiflones, 100) Bahía Mansa.

*Eupsophus emiliopugini* (map D): 64) P.N. Puyehue, 66) La Picada, 101) Lenca, 102) Camino a Maullín, 70) Guabún, 72) Puntra, 75) Cucao, 79) Yaldad, 103) Río Cisnes, 104) Isla Kent, 105) Caleta Vidal, 106) Isla Rivero.

*Eupsophus* sp. (maps A and B): 3) Cerro El Guanaco, 8) Cerros de Chiguayante, 9) Santa Juana, 10) Llico, 11) Quidico, 14) Alto Biobío, 15) Loncopangue, 18) Pemehue, 20) Río Traiguén, 24) Camino a Villa Las Araucarias, 27) Santa Amelia, 28) Pumalal, 31) Camino a P.N. Villarrica, 32) Malalhue, 42) Puringue, 56) Naguilán.

*Eupsophus* sp. 2 (map A): 19) P.N. Tolhuaca.

**Fig A. Historical and new localities of *Eupsophus* spp.**

Squares indicate localities included in the phylogenetic analysis (obtained for this study and from [18], Table 1). Circles indicate localities obtained from the literature, which were selected to represent the distribution ranges of species. For Argentina, only the two localities included in the present phylogenetic/delimitation analyses (from [18]) are shown. The size of symbols is proportional to the number of species present (1-3) in each locality, according to the cumulative information of the literature. Thin gray lines within Chile represent boundaries of Administrative Regions. (A-C) Localities of the *roseus* group. (D) Localities of the *vertebralis* group. See above the complete list of numbered localities.

**
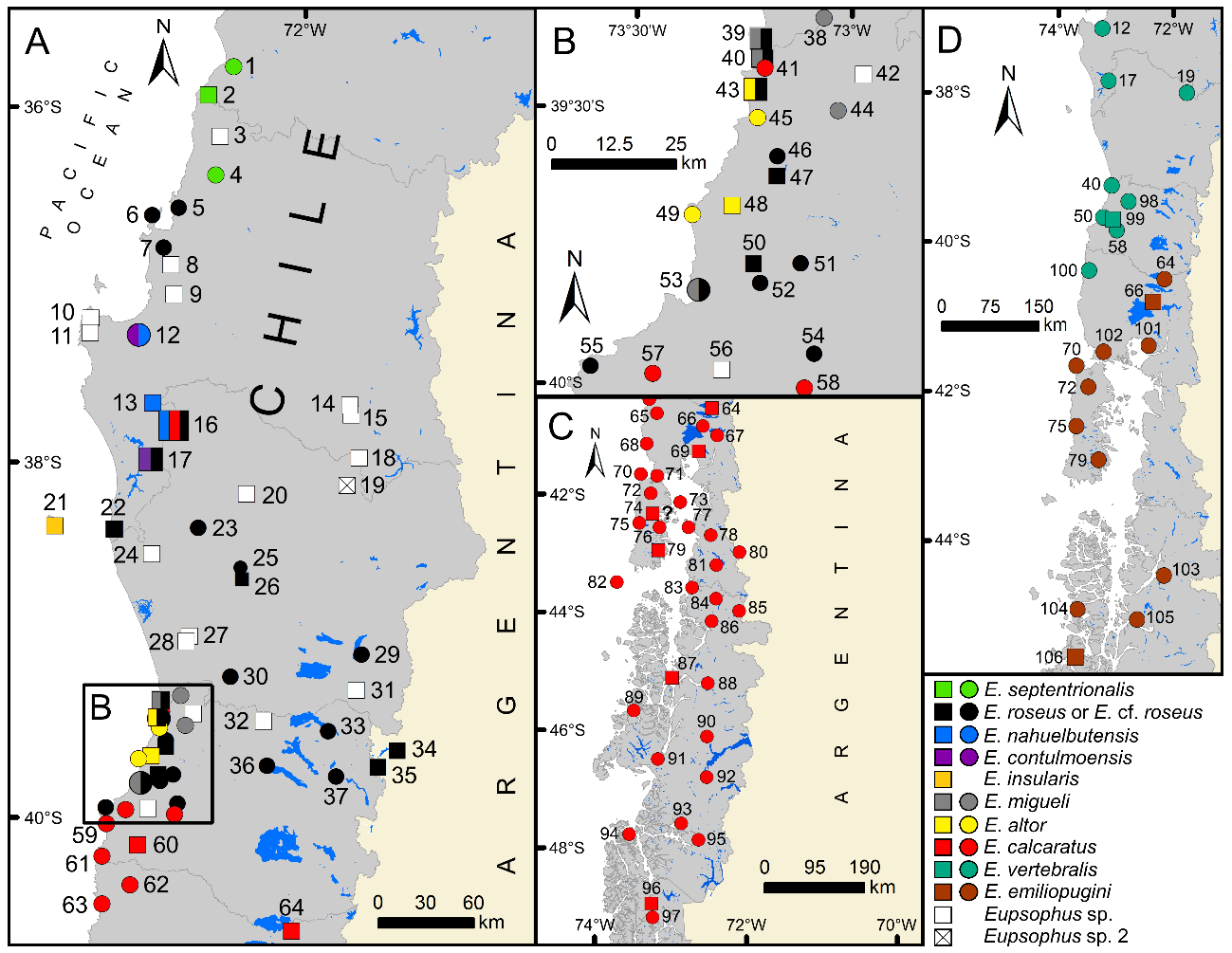
**
