# Supplementary material for: Species delimitation in frogs from South American temperate forests: The case of Eupsophus, a taxonomically complex genus with high phenotypic variation
Source: PLoS One. 2017 Aug 15;12(8):e0181026. doi: 10.1371/journal.pone.0181026 (PMC5557580; doi:10.1371/journal.pone.0181026)

S1 Fig. Examples of intrapopulation variation of diagnostic characters frequently used in the taxonomy of the *Eupsophus roseus* group (body coloration, snout profile and iris coloration; see details in S1 File). (A) Specimens from Camino a P.N. Villarrica (*E. roseus* according to our results) displaying different dorsal coloration patters, including one with a vertebral line; those patters have earned them the name of ground frogs. (B) Adult females from Valdivia, type locality of *E. roseus*; note the variation of body and iris coloration patterns, and of snout profile, ranging from short and rounded to long and pointed. (C) Specimens of *E. altor* from its type locality, Parque Oncol (*E. migueli* according to our results), showing the extensive variation of body coloration, iris coloration and design, and shape of snout. All these examples are based on no more of twelve specimens captured in a single session.


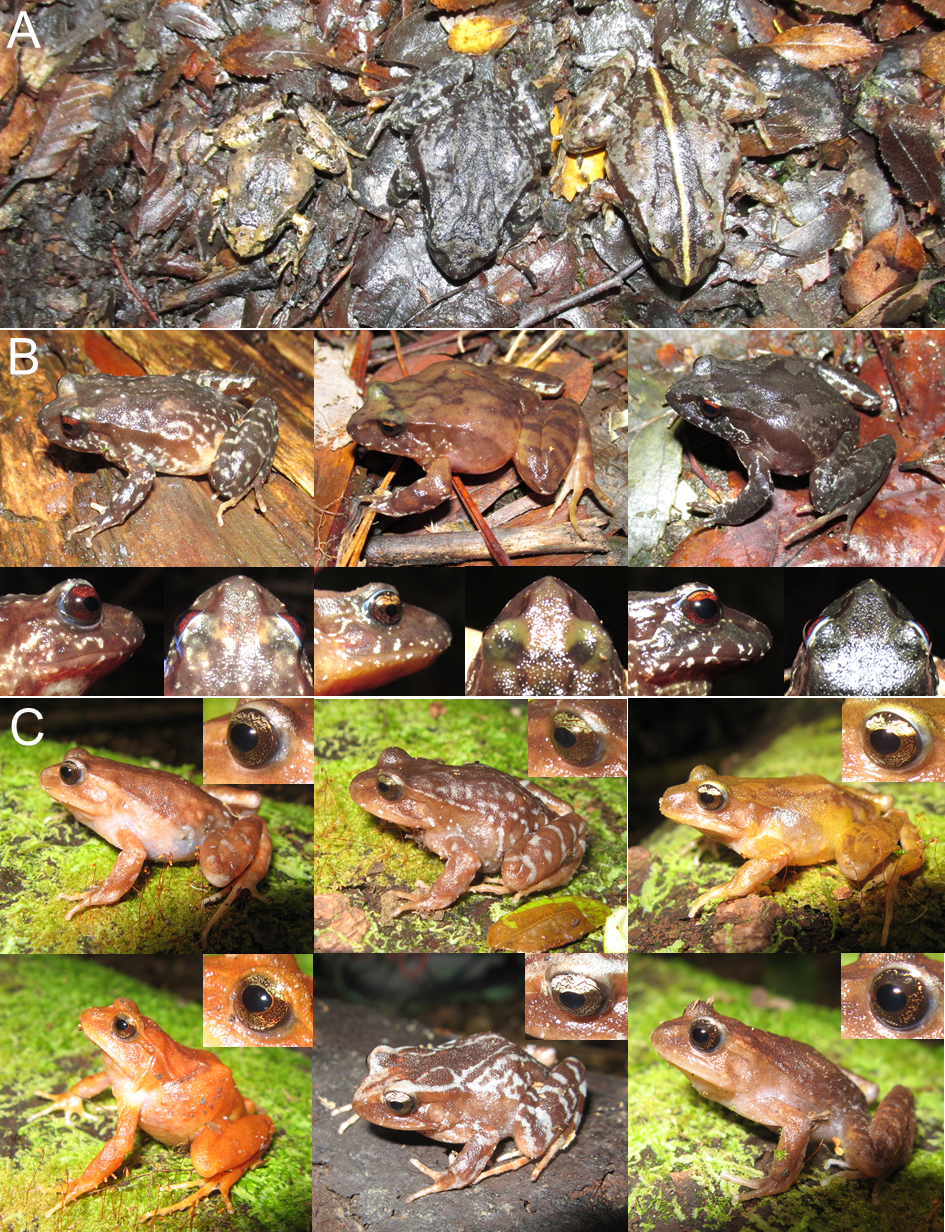

Supplement: S1 Fig — (DOCX) [file pone.0181026.s006.docx]
