# Supplementary material for: Species delimitation in frogs from South American temperate forests: The case of Eupsophus, a taxonomically complex genus with high phenotypic variation
Source: PLoS One. 2017 Aug 15;12(8):e0181026. doi: 10.1371/journal.pone.0181026 (PMC5557580; doi:10.1371/journal.pone.0181026)

S2 Fig. Additional examples of external phenotypic variation in two new populations of *Eupsophus roseus* (identification based on our species delimitation analyses)*.* (A) Two males from Santa Juana showing differences in body and iris coloration, and shape of snout. (B and C) Adults from Puringue showing variation in iris, dorsal and ventral coloration patterns.


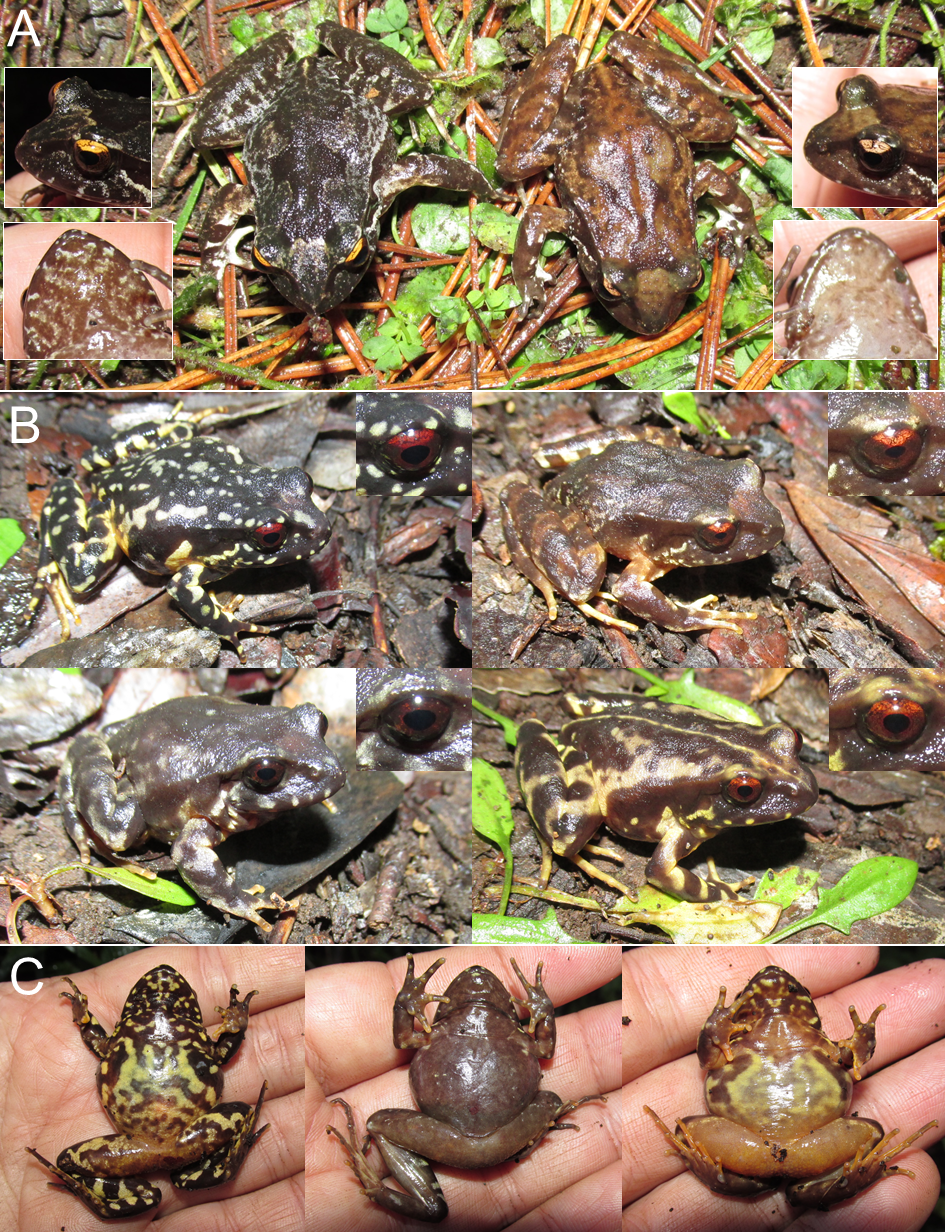

Supplement: S2 Fig — (DOCX) [file pone.0181026.s007.docx]
