# Supplementary material for: Species delimitation in frogs from South American temperate forests: The case of Eupsophus, a taxonomically complex genus with high phenotypic variation
Source: PLoS One. 2017 Aug 15;12(8):e0181026. doi: 10.1371/journal.pone.0181026 (PMC5557580; doi:10.1371/journal.pone.0181026)

**S3 Fig. Graphical results of the analysis ABGD.**

(A) Histogram of pairwise Kimura 2-parameter distances for the concatenated mitochondrial and nuclear sequences, showing two clear barcode gaps (blue arrows). (B) Ranked pairwise distances. (C) Number of groups for initial and recursive partitions per value of prior intraspecific divergence; arrows indicate what we consider the relaxed (seven groups) and conservative (four groups) results.


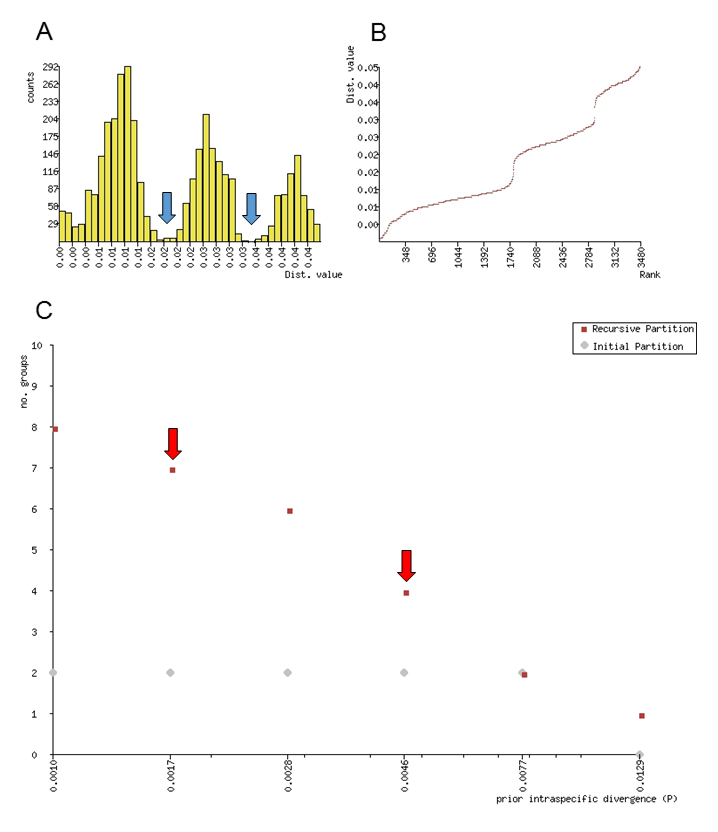

Supplement: S3 Fig — (DOCX) [file pone.0181026.s008.docx]
