# Supplementary material for: Species delimitation in frogs from South American temperate forests: The case of Eupsophus, a taxonomically complex genus with high phenotypic variation
Source: PLoS One. 2017 Aug 15;12(8):e0181026. doi: 10.1371/journal.pone.0181026 (PMC5557580; doi:10.1371/journal.pone.0181026)
Supplement: S1 Table — (DOCX) [file pone.0181026.s009.docx]

**S1 Table. Kimura two-parameter distances among the seven candidate species (CSs) obtained with mPTP and relaxed ABGD analyses.**

Values below the diagonal correspond to distances of mitochondrial sequences; values above the diagonal corresponds to those of mitochondrial plus nuclear sequences. Capital letters indicate the respective nodes of the trees of Figs 3 and 4. CSs are colored according to our conservative estimate of species of the *roseus* group (Fig 4). Note that distances of the mitochondrial plus nuclear data set between CS1 and CS2 (our *Eupsophus insularis*) and among CS5-CS7 (our *E. calcaratus*) are lower than the intra/interspecific threshold (0.25) inferred from the distribution of pairwise distances (S3 Fig).

|  | CS1 (D) | CS2 (C) | CS3 (A) | CS4 (E) | CS5 (G) | CS6 (I) | CS7 (H) |
| --- | --- | --- | --- | --- | --- | --- | --- |
| CS1 (D) | ̶ | 0,013 | 0,024 | 0,026 | 0,044 | 0,042 | 0,044 |
| CS2 (C) | 0,018 | ̶ | 0,025 | 0,029 | 0,046 | 0,045 | 0,047 |
| CS3 (A) | 0,032 | 0,034 | ̶ | 0,027 | 0,043 | 0,043 | 0,043 |
| CS4 (E) | 0,034 | 0,039 | 0,036 | ̶ | 0,043 | 0,041 | 0,043 |
| CS5 (G) | 0,060 | 0,063 | 0,059 | 0,058 | ̶ | 0,019 | 0,021 |
| CS6 (I) | 0,058 | 0,061 | 0,059 | 0,055 | 0,026 | ̶ | 0,013 |
| CS7 (H) | 0,059 | 0,063 | 0,058 | 0,058 | 0,028 | 0,017 | ̶ |
