# Supplementary material for: Species delimitation in frogs from South American temperate forests: The case of Eupsophus, a taxonomically complex genus with high phenotypic variation
Source: PLoS One. 2017 Aug 15;12(8):e0181026. doi: 10.1371/journal.pone.0181026 (PMC5557580; doi:10.1371/journal.pone.0181026)
Supplement: S2 Table — (DOCX) [file pone.0181026.s010.docx]

**S2 Table. Biological material to obtain DNA sequences in this study and GenBank accession numbers.**

The type of tissue used to obtain DNA is indicated: buccal mucosa (swab), in the case of the specimens released in situ, or muscle of tongue (tissue), in the case of the specimens deposited in collections as vouchers. DBGUCH: herpetological collection of the Departamento de Biología Celular y Genética of the Universidad de Chile; MZUC: Museo de Zoología of the Universidad de Concepción; SSUC-Am: Colección de Flora y Fauna Prof. Patricio Sánchez Reyes, Amphibian section, of the Departamento de Ecología, Pontificia Universidad Católica de Chile; IZUA: zoological collection of the Instituto de Zoología de la Universidad Austral. The codes that accompany the samples of the MZUC are provisional, since this collection is currently in reorganization. Swab and tissue samples are deposited in the collection of the Laboratorio de Herpetología, Biodiversidad y Ecología Molecular (LabHerp) of the Universidad de Concepción. Localities are ordered as in Table 1.

| Locality | Nominal species | Sample | Voucher/sample code | 12S-16S | rhod | SINA |
| --- | --- | --- | --- | --- | --- | --- |
| R.N. Los Queules | *E. queulensis* | tissue | DBGUCH 3311 | KY826246 | KY826306 | KY826366 |
| R.N. Los Queules | *E. queulensis* | tissue | DBGUCH 3312 | KY826247 | KY826307 | KY826367 |
| R.N. Los Queules | *E. queulensis* | tissue | DBGUCH 3313 | KY826248 | KY826308 | KY826368 |
| Cerro El Guanaco | *Eupsophus* sp. | tissue | MZUC EG0 | KY826249 | KY826309 | KY826369 |
| Cerros de Chiguayante | *Eupsophus* sp. | tissue | SSUC-Am223 | KY826250 | KY826310 | KY826370 |
| Cerros de Chiguayante | *Eupsophus* sp. | tissue | SSUC-Am224 | KY826251 | KY826311 | KY826371 |
| Cerros de Chiguayante | *Eupsophus* sp. | tissue | SSUC-Am225 | KY826252 | KY826312 | KY826372 |
| Santa Juana | *Eupsophus* sp. | tissue | SSUC-Am220 | KY826253 | KY826313 | KY826373 |
| Santa Juana | *Eupsophus* sp. | tissue | SSUC-Am221 | KY826254 | KY826314 | KY826374 |
| Santa Juana | *Eupsophus* sp. | tissue | SSUC-Am222 | KY826255 | KY826315 | KY826375 |
| Llico | *Eupsophus* sp. | tissue | MZUC Lli1 | KY826261 | KY826321 | KY826381 |
| Llico | *Eupsophus* sp. | tissue | MZUC Lli2 | KY826262 | KY826322 | KY826382 |
| Llico | *Eupsophus* sp. | tissue | MZUC Lli3 | KY826263 | KY826323 | KY826383 |
| Llico | *Eupsophus* sp. | tissue | MZUC Lli4 | KY826264 | KY826324 | KY826384 |
| Quidico | *Eupsophus* sp. | tissue | MZUC Qui1 | KY826265 | KY826325 | KY826385 |
| Quidico | *Eupsophus* sp. | tissue | MZUC Qui2 | KY826266 | KY826326 | KY826386 |
| Alto Biobío | *Eupsophus* sp. | tissue | MZUC ABB1 | KY826256 | KY826316 | KY826376 |
| Alto Biobío | *Eupsophus* sp. | tissue | MZUC BB1 | KY826257 | KY826317 | KY826377 |
| Loncopangue | *Eupsophus* sp. | tissue | MZUC Lon1 | KY826258 | KY826318 | KY826378 |
| P.N. Nahuelbuta | *E. nahuelbutensis* | tissue | MZUC Nah3 | KY826270 | KY826330 | KY826390 |
| P.N. Nahuelbuta | *E. nahuelbutensis* | tissue | SSUC-Am232 | KY826271 | KY826331 | KY826391 |
| P.N. Nahuelbuta | *E. nahuelbutensis* | tissue | SSUC-Am233 | KY826272 | KY826332 | KY826392 |
| M.N. Contulmo | *E. contulmoensis* | swab | LabHerp EcoMNC | KY826267 | KY826327 | KY826387 |
| M.N. Contulmo | *E. contulmoensis* | tissue | SSUC-Am199 | KY826268 | KY826328 | KY826388 |
| M.N. Contulmo | *E. contulmoensis* | tissue | SSUC-Am200 | KY826269 | KY826329 | KY826389 |
| Pemehue | *Eupsophus* sp. | tissue | MZUC Pem1 | KY826259 | KY826319 | KY826379 |
| Pemehue | *Eupsophus* sp. | tissue | MZUC Pem2 | KY826260 | KY826320 | KY826380 |
| Río Traiguén | *Eupsophus* sp. | swab | LabHerp CA1 | KY826273 | KY826333 | KY826393 |
| Río Traiguén | *Eupsophus* sp. | swab | LabHerp CA3 | KY826274 | KY826334 | KY826394 |
| Primer Agua | *Eupsophus* cf. *roseus* | tissue | MZUC PrA1 | KY826242 | KY826302 | KY826362 |
| Primer Agua | *Eupsophus* cf. *roseus* | tissue | MZUC PrA2 | KY826243 | KY826303 | KY826363 |
| Camino a Villa Las Araucarias | *Eupsophus* sp. | tissue | SSUC-Am190 | KY826244 | KY826304 | KY826364 |
| Camino a Villa Las Araucarias | *Eupsophus* sp. | tissue | SSUC-Am191 | KY826245 | KY826305 | KY826365 |
| M.N. Cerro Ñielol | *E. roseus* | swab | LabHerp Ñie1 | KY826275 | KY826335 | KY826395 |
| M.N. Cerro Ñielol | *E. roseus* | tissue | MZUC Ñie3 | KY826276 | KY826336 | KY826396 |
| M.N. Cerro Ñielol | *E. roseus* | tissue | MZUC Ñie4 | KY826277 | KY826337 | KY826397 |
| Santa Amelia | *Eupsophus* sp. | tissue | SSUC-Am216 | KY826281 | KY826341 | KY826401 |
| Santa Amelia | *Eupsophus* sp. | tissue | SSUC-Am217 | KY826282 | KY826342 | KY826402 |
| Pumalal | *Eupsophus* sp. | tissue | SSUC-Am205 | KY826283 | KY826343 | KY826403 |
| Camino a P.N. Villarrica | *Eupsophus* sp. | swab | LabHerp Vil1 | KY826278 | KY826338 | KY826398 |
| Camino a P.N. Villarrica | *Eupsophus* sp. | tissue | MZUC Vil2 | KY826279 | KY826339 | KY826399 |
| Camino a P.N. Villarrica | *Eupsophus* sp. | tissue | MZUC Vil3 | KY826280 | KY826340 | KY826400 |
| Mehuín | *E. migueli* | tissue | IZUA 3476 | KY826239 | KY826299 | KY826359 |
| Mehuín | *E. migueli* | tissue | MZUC Meh1h | KY826240 | KY826300 | KY826360 |
| Mehuín | *E. migueli* | tissue | MZUC Meh2 | KY826241 | KY826301 | KY826361 |
| Puringue | *Eupsophus* sp. | tissue | SSUC-Am207 | KY826284 | KY826344 | KY826404 |
| Puringue | *Eupsophus* sp. | tissue | SSUC-Am208 | KY826285 | KY826345 | KY826405 |
| Puringue | *Eupsophus* sp. | tissue | SSUC-Am209 | KY826286 | KY826346 | KY826406 |
| Malalhue | *Eupsophus* sp. | tissue | MZUC P9j | KY826287 | KY826347 | KY826407 |
| Parque Oncol | *E. altor* | swab | LabHerp PO1h | KY826236 | KY826296 | KY826356 |
| Parque Oncol | *E. altor* | tissue | MZUC PO2 | KY826237 | KY826297 | KY826357 |
| Parque Oncol | *E. altor* | tissue | MZUC PO3 | KY826238 | KY826298 | KY826358 |
| Valdivia | *E. roseus* | tissue | IZUA 3506 | KY826288 | KY826348 | KY826408 |
| Valdivia | *E. roseus* | tissue | SSUC-Am213 | KY826289 | KY826349 | KY826409 |
| Valdivia | *E. roseus* | tissue | SSUC-Am214 | KY826290 | KY826350 | KY826410 |
| Valdivia | *E. roseus* | swab | LabHerp LSh1 | KY826291 | KY826351 | KY826411 |
| Naguilán | *Eupsophus* sp. | tissue | MZUC Nag1 | KY826292 | KY826352 | KY826412 |
| Naguilán | *Eupsophus* sp. | tissue | MZUC Nag2 | KY826293 | KY826353 | KY826413 |
| Camino a P.N. Alerce Costero | *E. calcaratus* | tissue | MZUC Ale3 | KY826294 | KY826354 | KY826414 |
| Río Correntoso | *E. calcaratus* | tissue | DBGUCH 0902001 | KY826295 | KY826355 | KY826415 |
